# Identification of Pathogenic *Fusarium* spp. Causing Maize Ear Rot and Potential Mycotoxin Production in China

**DOI:** 10.3390/toxins8060186

**Published:** 2016-06-21

**Authors:** Canxing Duan, Zihui Qin, Zhihuan Yang, Weixi Li, Suli Sun, Zhendong Zhu, Xiaoming Wang

**Affiliations:** Institute of Crop Science, Chinese Academy of Agricultural Sciences/National Key Facility for Crop Gene Resources and Genetic Improvement/Cereal Quality Supervision and Testing Center, Ministry of Agriculture, Beijing 100081, China; duancanxing@caas.cn (C.D.); qinzihui0927@163.com (Z.Q.); zhihuan_yang@163.com (Z.Y.); liweixi@caas.cn (W.L.); sunsuli@caas.cn (S.S.); zhuzhendong@caas.cn (Z.Z.)

**Keywords:** maize, ear rot, *Fusarium* spp., mycotoxin chemotype, mycotoxin production

## Abstract

Ear rot is a serious disease that affects maize yield and grain quality worldwide. The mycotoxins are often hazardous to humans and livestock. In samples collected in China between 2009 and 2014, *Fusarium verticillioides* and *F. graminearum* species complex were the dominant fungi causing ear rot. According to the *TEF-1*α gene sequence, *F. graminearum* species complex in China included three independent species: *F. graminearum*, *F. meridionale*, and *F. boothii*. The key gene *FUM1* responsible for the biosynthesis of fumonisin was detected in all 82 *F. verticillioides* isolates. Among these, 57 isolates mainly produced fumonisin B_1_, ranging from 2.52 to 18,416.44 µg/g for each gram of dry hyphal weight, *in vitro*. Three different toxigenic chemotypes were detected among 78 *F. graminearum* species complex: 15-ADON, NIV and 15-ADON+NIV. Sixty and 16 isolates represented the 15-ADON and NIV chemotypes, respectively; two isolates carried both 15-ADON and NIV-producing segments. All the isolates carrying NIV-specific segment were *F. meridionale*. The *in vitro* production of 15-ADON, 3-ADON, DON, and ZEN varied from 5.43 to 81,539.49; 6.04 to 19,590.61; 13.35 to 19,795.33; and 1.77 to 430.24 µg/g of dry hyphal weight, respectively. Altogether, our present data demonstrate potential main mycotoxin production of dominant pathogenic *Fusarium* in China.

## 1. Introduction

*Fusarium* spp. are among the most important pathogenic fungal communities affecting crops. These fungi can induce serious diseases on roots, stems, leaves, and fruits, resulting in huge economic losses. Maize ear rot caused by *Fusarium* spp. affects maize production and kernel quality. The disease occurs in many countries; in recent years, it has gradually expanded because of global warming [1,2,3]. In addition to causing ear rot and reducing yield, *Fusarium* spp. produce mycotoxins that are directly synthesized in kernels and accumulated there, hence, seriously threatening the health of both humans and livestock [4,5,6]. The most common *Fusarium* toxins are deoxynivalenol (DON) and the fumonisins. They are very often detected in cereal crops such as maize, wheat, and rice and have drawn wide attention owing to their toxicity and carcinogenicity. In 1973, *Fusarium* toxin was listed among the 16 research priorities of the World Health Organization and the Food and Agriculture Organization [7,8,9,10]. With improvements in living standards, higher requirements for food safety have been proposed.

*Fusarium verticillioides* is one of the most common pathogens causing ear rot in maize. This disease is widespread in temperate and semitropical areas, including all European maize-growing areas, and even prevailing in Mediterranean areas, such as the agricultural regions of Italy, which are characterized by dry, warm conditions [3,11]. In recent years, *F. verticillioides* has also been characterized as an important pathogen causing maize ear rot in several regions of China [12,13,14]. This fungus is able to produce dangerous fumonisins that can be toxic for humans and animals. The fumonisins have been classified into four main groups: fumonisins A, B, C, and P. The fumonisin B (FB) analogs—comprising toxicologically important FB_1_, FB_2_, and FB_3_—are the most abundant naturally-occurring fumonisins. FB_1_ is the most toxic and appears in the highest concentration in the host, accounting for 70% to 80% of total fumonisins [5,15,16]. This mycotoxin can be carcinogenic in humans and livestock and even cause equine leukoencephalomalacia, rat hepatocarcinoma, and porcine pulmonary edema [3,7].

Production of fumonisins by *F. verticillioides* is dependent on a biosynthetic gene cluster (*FUM*) made up of 16 contiguous and coexpressed genes; deletion of the gene *FUM1* can reduce FB_1_ production by 99% [17,18]. The various toxicological profiles of *F. verticillioides* isolates may reflect significant differences in the risk for mycotoxin contamination, with potential implications for human and animal health and international trade [19].

*Fusarium graminearum* species complex (FGSC) comprises a class of important pathogens of small-grain cereals and maize in many areas of the world. These fungi often cause economically devastating diseases that occur at different stages of plant development, such as seedling blight, maize root rot, wheat head blight (FHB or scab), and maize ear rot, causing a significant eventual reduction in the quality and yield of the crops [20,21,22].

Moreover, these diseases are often associated with various trichothecenes and other mycotoxins produced in plants, thus representing an important problem of food safety. FGSC can produce zearalenone (ZEN), nivalenol (NIV), and DON, which can inhibit protein synthesis by combining with the 60S ribosomal subunit. This poses a significant threat to the health of both humans and livestock [5,23]. *Fusarium* spp. can produce many types of trichothecenes. Type B trichothecenes are among the mycotoxins produced by the FGSC. The particularly important groups within this type are DON and its acetylated derivatives, 3-acetyl-deoxynivalenol (3-ADON) and 15-acetyl-deoxynivalenol (15-ADON), as well as nivalenol (NIV) and its acetylated derivatives, 4-acetylnivalenol. 3-ADON has been shown to be more phytotoxic and fungi that produce 3-ADON have a higher pathogenic potential than those producing 15-ADON [23,24,25].

Among specimens of *F. graminearum* isolated from barley, wheat, potato, and beet in America, 15-ADON–producing isolates were more common than those producing 3-ADON, and NIV was detected only in the isolate number 21 [26,27]. In North America, 3-ADON–producing isolates have been found to produce a higher DON content, and these may be replacing 15-ADON isolates with weaker pathogenicity [28,29]. In northern Europe and northwestern Russia, more 3-ADON isolates were present as compared with 15-ADON isolates [30,31,32]. The mycotoxins 15-ADON and NIV were produced by *F. graminearum* in Luxembourg and 15-ADON was the dominant type, while no 3-ADON–producing isolates were detected [33]. Of 42 *F. culmorum* isolates collected in Australia and nine European countries, 34 isolates produced DON and seven produced high concentrations of NIV but low levels of DON, while one produced neither DON nor NIV [34]. A recent study showed that in *F. graminearum* collected from Europe, the predominant genotype was 15-ADON (82.9%), followed by 3-ADON (13.6%), and nivalenol (NIV) (3.5%). In *F. culmorum*, the prevalent genotype was 3-ADON (59.9%), while the NIV genotype accounted for 40.1% [35].

Many methods can be used to detect *Fusarium* mycotoxins, including enzyme-linked immunosorbent assay, thin-layer chromatography, capillary electrophoresis, gas chromatography, gas chromatography/mass spectrometry, high-performance liquid chromatography (HPLC), liquid chromatography/mass spectrography, and ultra-high-performance liquid chromatography/mass spectrometry (UHPLC-MS/MS). HPLC and UHPLC-MS/MS are widely used in quantitative assays of mycotoxin because of their high efficiency and accuracy [36,37,38,39].

A good knowledge of the potential for mycotoxin biosynthesis by fungal isolates from different geographic regions could help to predict the risk of contamination by mycotoxins in the surveyed areas. To date, very little information on chemotypes and the mycotoxin-producing capacity of the main pathogenic *Fusarium* spp. causing maize ear rot in China has been reported. In the research documented here, samples were collected from the main maize producing areas experiencing frequent ear rot; pathogenic *Fusarium* spp. were then isolated and identified. Mycotoxin chemotypes and their production were examined. The aim was to determine the pathogenic species of *Fusarium* causing maize ear rot in China, as well as the main mycotoxin chemotypes and their production capacity.

## 2. Results

### 2.1. Identification of *Fusarium* spp.

**Figure 1:**
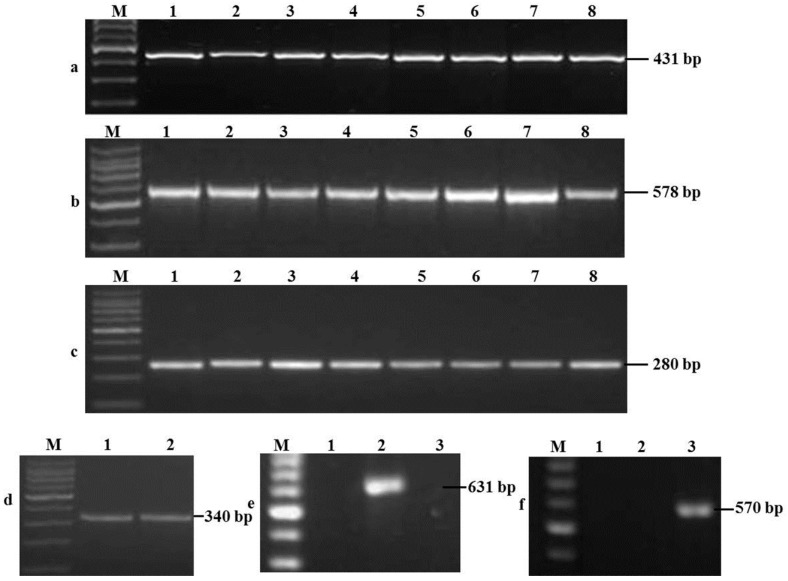
Molecular detection of *Fusarium* species. M, DNA marker; (**a**) *Fusarium* spp. were specifically amplified by polymerase chain reaction (PCR), 1–8: *Fusarium* spp. FG029, FG030, FG039, FG042, FG044, FVAH-1, FVAH-3, FVHB-4; (**b**) PCR amplification of *F. verticillioides*, 1–8: *F. verticillioides* FVAH-1 FVAH-3, FVHB-4, FVHB-5, FVHB-6, FVHN-7, FVHN-8, FVSD-9; (**c**) PCR amplification of *F. graminearum* species complex, 1–8: FGSC FG029, FG030, FG039, FG042, FG015, FG020, FG092, FG146; (**d**) PCR amplification of *F. oxysporum* species complex, 1–2: *F. oxysporum* species complex HN1, AH1; (**e**) PCR amplification of *F. subglutinans*, 2: *F. subglutinans* S’XX; 1, 3: *F. verticillioides* FVHB-4, FVHB-5; and (**f**) PCR amplification of *F. culmorum*, 1–2: FGSC FG080, FG081, 3: *F. culmorum* SD1.

According to morphological and molecular findings, 160 tested maize seed samples, accounting for 66.9% of total samples, were infected by *Fusarium* spp. and most of them were *F. verticillioides* and FGSC. 82 *F. verticillioides* isolates with the specific amplicon of 578 bp size were obtained from 82 samples (51.3%) at 67 places in 18 provinces, whereas FGSC was isolated from 70 samples (43.8%) at 66 places in 17 provinces. In addition, *F. culmorum*, *F. oxysporum*, *F. proliferatum*, *F. subglutinans*, and *F. solani* clades were isolated from three (1.9%), two (1.3%), one (0.6%), one (0.6%), and one (0.6%) sample, respectively (Figure 1).

**Figure 2:**
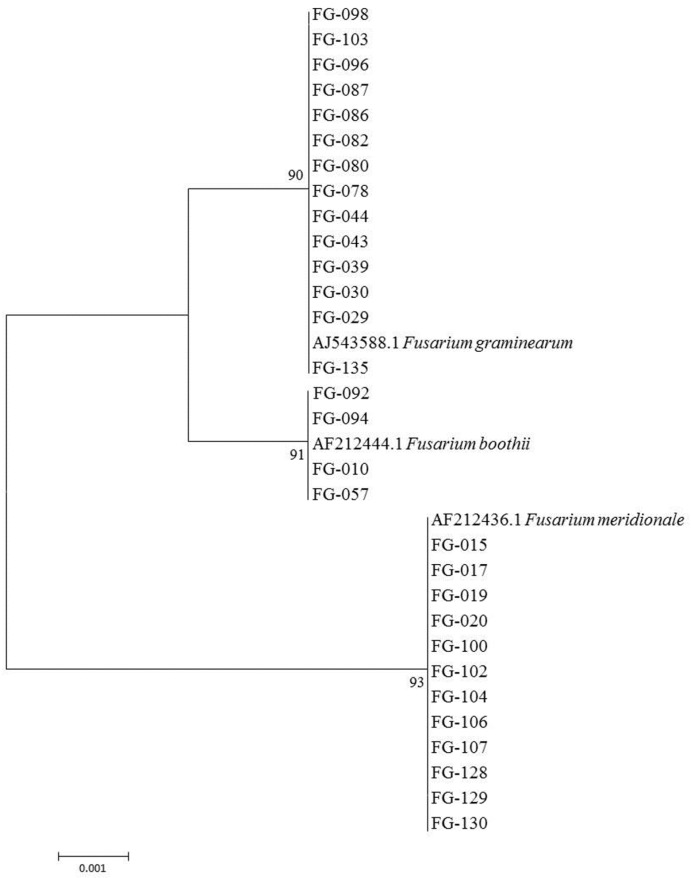
The phylogenetic tree of some *F. graminearum* species complex based on *TEF-1*α gene sequences. Branch lengths are proportional to the estimated number of nucleotide substitutions. The BP values are displayed on the nodes (BP: 1000 replicates).

Sequencing of the *TEF-1*α gene of 78 FGSC isolates and alignment with BLAST in the Fusarium Center’s database showed 99% to 100% homology between the tested FGSC and standard reference strains in this database. A total of 42 isolates—such as FG-029, FG-30, FG-043, FG-103 and others—exhibited 99% to 100% homology with *F. graminearum* sensu stricto (AJ543588.1), while the other 20 isolates (e.g., FG-015, FG-020, FG-100, FG-130, and the like) showed 99% to 100% homology with *F. meridionale* (AF212436.1). In addition, 16 isolates (e.g., FG-010, FG-057, and FG-094) expressed 100% homology with *F. boothii* (AF212444.1). The phylogenetic tree of some FGSC based on *TEF-1*α gene sequences confirmed *Fusarium* species. The tree topologies of the *TEF-1*α gene sequences showed the classification of FGSC into three distinct clades and the support values for these clades were high (no lower than 90% bootstrap value; BP) (Figure 2). These results demonstrated that the FGSC causing maize ear rot in China is mainly *F. graminearum*, which accounted for 53.8% of the total FGSC and was widely distributed in northern and southern maize-producing regions. The second main lineage was *F. meridionale*, accounting for 25.6% of total FGSC. This lineage was isolated only from Yunnan, Guizhou, and southern Shaanxi provinces. A total of 16 isolates showed 100% homology with *F. boothii*, accounting for 20.5% of the total; these were discovered in northern areas of China (e.g., Inner Mongolia, Jilin, Hebei, Shanxi, and Beijing).

### 2.2. Detection of Toxigenic Genes and Chemotypes

*FUM1* was detected in 82 *F. verticillioides* isolates, thus indicating that these isolates can potentially synthesize FBs.

Three different mycotoxin chemotypes were detected from 78 isolates in FGSC. In all, 60 isolates with the specific 583-bp amplified fragments were 15-ADON, while 16 isolates producing the specific 859-bp amplified fragments were NIV. In addition, 583- and 859-bp amplified fragments produced by two isolates simultaneously were 15-ADON and NIV.

According to the molecular identification of NIV, 15-ADON, and 3-ADON of FGSC, 62 FGSC isolates collected from Beijing, Hebei, Liaoning, Jilin, Heilongjiang, Inner Mongolia, Henan, Shandong, Anhui, Ningxia, and Gansu were detected with only 15-ADON. Those in Guizhou were detected with only NIV. Both 15-ADON- and NIV-producing isolates were detected in samples from Shaanxi Province. Samples collected in Shaanxi Province had the most mycotoxin types. Isolates from northern Shaanxi had two main mycotoxin types, but 15-ADON was dominant in isolates from Guanzhong and northern Shaanxi Province. All 16 independent NIV isolates were identified as *F. meridionale* and mainly originated from Southwestern China. The two isolates containing specific 15-ADON- and NIV-producing segments were from southern Shaanxi.

### 2.3. Detection of Toxigenic Capacity

The results of FB detection with immunoafinity chromatography purification-HPLC and DON and ZEN assays based on UPLC-MS/MS agreed with the molecular identification of key mycotoxin-producing genes.

A fumonisin assay showed that the mycotoxin-producing capacity of *F. verticillioides* isolates from different regions varied greatly and that FB_1_ was the main chemotype. Among 82 *F. verticillioides* isolates, 79, 69, and 75 isolates produced FB_1_, FB_2_, and FB_3_ respectively. A total of 70 isolates were able to produce FB_1_, FB_2_, and FB_3_ simultaneously, and 12 isolates produced no FB_2._ Among the latter, seven produced no FB_3_. Among isolates producing FB, 57 isolates produced mainly FB_1_ and 23 produced mainly FB_2_ or FB_3_. The production capacities of FB_1_, FB_2_, and FB_3_ ranged from 2.52 to 18,416.44, 3.00 to 1864.90, and 0.28 to 1510.54 µg/g of dry hyphal weight, respectively (Table 1).

Among FGSC, 15-ADON was detected in 68 isolates, showing a mycotoxin production range of 5.43 to 81,539.49 µg/g of dry hyphal weight. Among 10 isolates producing no 15-ADON, eight were *F. meridionale*, which produced NIV according to molecular identification; they were mainly distributed in Hubei, Sichuan, and Bijie of Guizhou. A total of 22 isolates had a low mycotoxin-producing capacity (≤1000 µg/g of dry hyphal weight). Among these 22 isolates, 12 were *F. meridionale*, including 11 NIV isolates from Guizhou, Yunnan, and Shangluo in southern Shaanxi Province. A total of 26 isolates had a moderate mycotoxin-producing capacity (from 1001 to 10,000 µg/g of dry hyphal weight). All of these were *F. graminearum* and *F. boothii*. Meanwhile 20 isolates had a high mycotoxin-producing capacity (>10,000 µg/g of hypha), all of which were *F. graminearum* and *F. boothii* (Table 2).

3-ADON was detected in 65 isolates, which showed a mycotoxin-producing capacity ranging from 6.04 to 19,590.61 µg/g of dry hyphal weight. Among 13 isolates producing no 3-ADON, 10 were *F. meridionale* and eight were NIV-producing isolates. They were all distributed in Hubei, Sichuan, Guizhou, and Yunnan. A total of 23 isolates had a low mycotoxin-producing capacity (≤1000 µg/g of hypha), including 10 *F. meridionale* isolates, while 39 isolates had a moderate mycotoxin-producing capacity (1001–10,000 µg/g of dry hyphal weight), including 27 *F. graminearum* and 12 *F. boothii* isolates. Three isolates with a high mycotoxin-producing capacity (>10,000 µg/g of dry hyphal weight) were *F. graminearum*.

DON was detected in 51 isolates, which showed a mycotoxin-producing range of 13.35 to 19,795.33 µg/g of dry hyphal weight. A total of 27 isolates, including 21 *F. meridionale*, 4 *F. graminearum*, and two *F. boothii* isolates, did not produce DON. A total of 16 isolates had a low mycotoxin-producing capacity (≤1000 µg/g of hypha), whereas 31 isolates had a moderate mycotoxin-producing capacity (1001–10,000 µg/g of dry hyphal weight), including 20 *F. graminearum* and 11 *F. boothii* isolates. Meanwhile, four *F. graminearum* isolates had a high mycotoxin-producing capacity (>10,000 µg/g of dry hyphal weight) (Table 2).

ZEN was detected in 24 isolates, which showed a mycotoxin-producing capacity varying from 1.77 to 430.24 µg/g of dry hyphal weight. Of these isolates, 13 were *F. graminearum*, nine were *F. boothii*, and two were *F. meridionale*. However, 54 isolates did not produce ZEN (Table 2).

Overall, *F. graminearum* had the highest mycotoxin-producing capacity, followed by *F. boothii* and *F. meridionale* successively in the laboratory. *F. graminearum* and *F. boothii* isolates with a high mycotoxin producing capacity were distributed mainly in Northern China.

Isolates with different chemotypes produced different amounts of mycotoxin. According to molecular identification and mycotoxin assay, 15-ADON–producing isolates produced more 15-ADON than DON and 3-ADON. None of the 18 isolates carrying NIV-specific segment produced any DON and 16 of them did nor ZEN. Most NIV-producing isolates produced more 3-ADON than 15-ADON. Also, the isolates carrying both 15-ADON and NIV chemotype-specific segments produced more 3-ADON than 15-ADON (Table 2). As a result, the isolates with NIV chemotype-specific segments likely pose a greater risk to maize crops as well as to humans and livestock because 3-ADON is more toxic than 15-ADON.

## 3. Discussion

Many pathogenic *Fusarium* spp. can be found causing maize ear rot in China, but the majority of isolates are determined to be *F. verticillioides* and FGSC. According to the frequency of the isolation of these species from different regions, maize ear rot is most commonly caused by *F. verticillioides*, followed by FGSC. This result is similar to that reported by other previous studies of the predominant pathogens causing ear rot in China, but compared with the regional research, there are also differences [12,13,40].

Maize samples infected by *F. verticillioides* are often contaminated with FBs. With regard to the levels of fumonisins produced by the isolates, three of 82 isolates (0.037%) were unable to produce fumonisins, indicating the presence of phenotypic variability among the *F. verticillioides* isolates. Interestingly, *FUM1*, a key gene responsible for FB biosynthesis, was detected in all 82 isolates. This result was similar to that of previous studies. Sanchèz-Rangel *et al.* reported that some isolates of *F. verticillioides* from maize in Mexico possessed the *FUM1* gene but did not produce fumonisins [41]. Several *F. verticillioides* isolates from Italy also carried the *FUM1* gene but did not produce fumonisins [3]. These phenotypes could be explained by a mutation in the *FUM* cluster or by altered expression of the *FUM* genes.

In this study, the toxigenic capacity of isolates from different regions varied, indicating that under conditions of laboratory incubation the mycotoxin chemotype and toxigenic capacity of *F*. *verticillioides* may not be correlated with its region of origin. Actually, in addition to the particular isolate, mycotoxin synthesis in plants also depends heavily on environment (temperature, humidity, pH, and lighting) as well as crop rations in the field. Therefore, mycotoxin production in the laboratory primarily represents toxigenic potentiality.

The FGSC can cause various gramineous crops diseases and leads to a reduction in crop yield and quality. In recent years, a total of 16 species in FGSC have been identified [42,43,44]. In the present study, three species lineages were found in the FGSC isolates collected mainly from Northeast China, Huang-Huai-Hai region, and Southwest China, regions in which the environments vary greatly.

In the present study, a total of 3 toxigenic chemotypes produced by FGSC were detected and DON was the main one. All *F. graminearum* and *F. boothii* isolates were 15-ADON–producing isolates, but *F. meridionale* produced one or more isolates of 15-ADON, NIV, and 3-ADON, which indicated that lineage or species was related to the mycotoxin chemotype to a certain extent. The genetic identification (molecular identification) of 10 isolates disagreed with the UHPLC assay: four were 15-ADON isolates (FG039, FG147, FG151, and FG152) and six were NIV isolates (FG017, FG140, FG142, FG143, FG145, and FG146). No mycotoxin had been detected by UHPLC in these isolates. In addition, based on the UHPLC quantitative assay of mycotoxin production, some amount of DON, 3-ADON, or ZEN was detected in 15-ADON–producing isolates and some 15-ADON or 3-ADON was detected in NIV-producing isolates. This outcome may be related to the expression and regulation of the toxin gene.

In the present study, 15-ADON–producing isolates generated more 15-ADON than 3-ADON, whereas NIV-type isolates generated more 3-ADON than 15-ADON, which may be related to the prior expression of a certain mycotoxin. Some DON-producing isolates coproduced a certain amount of NIV, as observed by Sugiura *et al*. [45].The mycotoxin production varied greatly among isolates from different regions. The occurrence of *Fusarium* spp. isolates with strong toxicity or high mycotoxin production should be given particular attention. Furthermore, two isolates were found to carry both DON and NIV chemotype-specific segments, possibly resulting from recombination between the two chemotypes. Kim *et al.* reported that a *F. graminearum* isolate (A18) from corn generated a chimeric pattern containing features of both DON-specific and NIV-specific sequences [46], which was similar to FG102 and FG105. With regard to the isolates carrying diverse chemotype-specific regions, special care must be taken, since these isolates have the potential to produce a variety of mycotoxins.

In summary, *F. verticillioides* and FGSC were the predominant fungi causing maize ear rot in China and the latter contained *F. graminearum*, *F. meridionale*, and *F. boothii*. Most of the *F. verticillioides* isolates mainly produced highly toxic fumonisin B_1_. The analysis of the current *Fusarium* chemotypes showed that trichothecene genotype was 15-ADON in all *F. graminearum* and *F. boothii* isolates. The prevalent chemotype was NIV (76.2%), followed by 15-ADON (14.3%) and NIV + 15-ADON (9.5%) in *F. meridionale*. The toxogenic capacity of *F. graminearum* and *F. boothii* was higher than that of *F. meridionale.*

## 4. Materials and Methods

### 4.1. Sample Collection and Isolation

A total of 239 samples of maize ears or kernels showing ear rot (five affected maize ears or 500 g kernels for each sample) were collected from 110 counties and districts of 18 provinces in China between 2009 and 2014 (Table 3). All samples were collected from different villages or fields of different towns, counties (districts), cities, and provinces (municipalities). Approximately 20 seeds collected from the boundary between maize ear rot regions and healthy regions or 90 seeds (30 seeds for each petri dish) from 500 g of kernels sample were soaked in 20% sodium hypochlorite solution for three minutes and rinsed with sterile water three times. These seeds were dried with sterilized filter paper and placed on a poor-nutrient potato dextrose agar (half-PDA) (potato infusion 100 g, dextrose 20 g, agar 20 g, distilled water 1000 mL) plate to culture for three days at 25°C. Hyphae or colonies on half-PDA with the morphological characteristics of *Fusarium* were transferred to fresh media and the culture was subsequently purified for 5 to 7 days. Hyphae were inoculated onto Spezieller Nährstoffarmer Agar (SNA) medium and incubated for seven days [47]. After conidia were generated, a single spore was isolated on SNA medium by the plate dilution method. Finally, the single spore was transplanted onto a half-PDA plate to culture single-spore isolates.

### 4.2. Identification of Pathogenic Fungi

The morphological identification of fungal cultures was conducted based on conidial morphology [47]. Subsequently, in order to confirm the morphological identification, all isolates were validated by species-specific PCR identification.

Mycelia were harvested by filtration through a filtering cloth, freeze-dried for 24 h, and then stored at −80 °C. The total genomic DNA of isolates was extracted from the mycelium, as described by Liu *et al.* [48]. Molecular identification was based on specific primers of different *Fusarium* spp. Primer sequences are listed in Table 4. The polymerase chain reaction (PCR) was carried out in a 20.0-µL receptacle containing 10 mM Tris-HCl pH 8.3, 50 mM KCl, 1.5 mM MgCl_2_, 0.5 mM of each dNTP, 2.0 µM of each primer, 1.0 U Taq polymerase (Dingguo, Beijing, China), and 50 ng of DNA template. Reactions were performed using a GeneAmp PCR System 9700 thermal cycler (ABI, Norwalk, CT, USA) programmed for 94 °C for 4 min; followed by 35 cycles of 94 °C 40 s, 57 °C 40 s (56 °C for *F. verticillioides*), and 72 °C 40 s; and a final extension at 72 °C for 10 min.

The translation elongation factor (*TEF*)*-1*α gene sequences of FGSC isolates was analyzed. Isolates that had been determined as FGSC were further identified by the sequences of the *TEF-1*α gene, with primers of EF-1 (5′-ATGGGTAAGGARGACAAGAC-3′) and EF-2 (5′-GGARGTACCAGTSATCATGTT-3′) [53]. FGSC isolates were amplified at a 50.0-µL reaction system containing 10 mM Tris-HCl pH 8.3, 50 mM KCl, 1.5 mM MgCl_2_, 0.5 mM of each dNTP, 2.0 µM of each primer, 1.0 U Taq polymerase, and 50 ng of DNA template. PCR was performed using the following parameters: 94 °C for 4 min; followed by 35 cycles of 94 °C 30 s, 53 °C 30 s, and 72 °C for 1 min, and a final extension at 72 °C for 10 min. To determine *Fusarium* species in FGSC, the sequences of the *TEF-1*α gene were compared with *Fusarium* sequences in the Fusarium Center’s database at Penn State [54]. Three standard strains were used as references to carry on homology BLAST (Table 5). Furthermore, using MEGA 5.0 software (ASU, Phoenix, Az, USA, 2011), a phylogenetic tree was constructed using the ML (Test Maximum Likelihood Tree) clustering method based on the *TEF-1*α gene sequences of FGSC isolates and standard reference strains (Figure S1) and the bootstrap analysis was performed with 1000 replicates for statistical support of branches [55].

### 4.3. Molecular Identification of Toxigenic Genes

Based on the molecular identification of all *F. verticillioides* isolates, the key gene *FUM1* responsible for FB biosynthesis was detected by using the specific primers Fum5F (5′-GTCGAGTTGTTGACCACTGCG -3′) and Fum5R (5′-CGTATCGTCAGCATGATGTAGC -3′) [49]. The PCR reaction was performed in a 20.0-µL receptacle containing 10 mM Tris-HCl pH 8.3, 50 mM KCl, 1.5 mM MgCl_2_, 0.5 mM of each dNTP, 2.0 µM of each primer, 1.0 U Taq polymerase, and 50 ng of DNA template. Reactions were programmed for 94 °C for 4 min; followed by 35 cycles of 94 °C 40 s, 60 °C 40 s, and 72 °C 1 min; and a final extension was performed at 72 °C for 10 min.

A mycotoxin-producing gene *Tri13* of FGSC was detected with primer Tri13P1/2. Primer sequences were *TRi*13P1 (5′-CTCSACCGCATCGAAGASTCTC-3′) and Tri13P2 (5′-GAASGTCGCARGACCTTGTTTC-3′). The PCR system and program were the same as above except for the annealing temperature of 58 °C. The amplified fragments of isolates producing NIV, 3-ADON, and 15-ADON were 859, 644, and 583 bp, respectively [56].

### 4.4. HPLC Detection of Mycotoxin Production

Equivalent *Fusarium* spp. were cut from half-PDA and placed in a sterilized conical flask with 150 mL of potato dextrose broth (PDB). Three flasks for each fungal isolate were inoculated. *F. graminearum* was grown in a 15-day shaking culture (120 rpm) in PDB with pH 3.0 at 25 °C. *F. verticillioides* was grown in a 15-day static culture in PDB with pH 8.0 at 25 °C [3,36]. The culture medium was filtered through Whatman GF/A glass fiber filter paper; filtrate was then stored at −80°C or sterilized under high pressure. Hyphae were collected, dried, and weighed. DON and ZEN production of FGSC was tested by UHPLC-quadrupole mass spectrometry. Samples were extracted by 80% acetonitrile water solution, purified via a multifunction decontamination column, isolated via a Waters ACQUITY UPLCTMBEH C_18_ chromatographic column, tested by multireaction ion monitoring of quadrupole mass spectrometry, and quantified by an external standard method [38]. The FBs production capacity of *F. verticillioides* was tested by immunoaffinity column purification/HPLC. The samples were purified with an immunoaffinity column to collect eluent. The eluent was dried with nitrogen and dissolved in 1.5 mL of 80% methanol solution. FBs were tested through a C_18_ reverse-phase liquid chromatography/fluorescence detector after o-phthaldialdehyde (OPA) derivation and quantified via an external standard method [36]. Statistical analysis was performed with SPSS10.0 software (SPSS Inc., Chicago, IL, USA, 2007).

## Figures and Tables

**Table 1:** Mycotoxin production of *Fusarium verticillioide* isolates.

No.	Origin of Isolate	FB_1_ (µg/g)	FB_2_ (µg/g)	FB_3_ (µg/g)	FB (µg/g)
FVAH-1	Sixian, Anhui	3437.79 ± 39.21	253.12 ± 11.23	69.91 ± 2.66	3760.82 ± 51.21
FVAH-2	Wuhe, Anhui	533.71 ± 15.32	133.42 ± 8.11	27.90 ± 3.58	695.04 ± 15.22
FVAH-3	Xiaoxian, Anhui	695.72 ± 13.89	76.33 ± 6.23	30.18 ± 1.56	802.24 ± 13.56
FVAH-4	Sixian, Anhui	4837.44 ± 56.66	248.03 ± 7.89	121.23 ± 8.44	5206.70 ± 56.67
FVAH-5	Suzhou, Anhui	1427.11 ± 20.13	150.57 ± 5.24	31.67 ± 2.31	1609.35 ± 22.14
FVAH-6	Suzhou, Anhui	1605.32 ± 23.25	170.88 ± 4.61	44.21 ± 2.85	1820.41 ± 20.41
FVBJ-1	Changping, Beijing	123.00 ± 9.85	48.18 ± 2.12	8.00 ± 1.13	179.18 ± 13.01
FVGS-1	Zhenyuan, Gansu	1750.53 ± 15.22	144.26 ± 3.43	25.77 ± 1.89	1920.56 ± 15.21
FVGZ-1	Bijie, Guizhou	2432.10 ± 13.85	172.07 ± 12.88	27.01 ± 2.14	2631.18 ± 23.33
FVGZ-2	Xifeng, Guizhou	14.06 ± 1.31	33.46 ± 2.56	24.51 ± 0.92	72.03 ± 5.85
FVGZ-3	Xifeng, Guizhou	12.58 ± 1.02	30.12 ± 1.78	21.87 ± 088	64.57 ± 4.12
FVGZ-4	Qianxinan, Guizhou	14.29 ± 1.15	31.27 ± 1.36	22.91 ± 3.11	68.47 ± 3.66
FVHB-1	Shenzhou, Hebei	40.04 ± 2.89	14.10 ± 2.11	47.26 ± 0.98	101.39 ± 10.23
FVHB-2	Hengshui, Hebei	226.85 ± 8.87	71.20 ± 5.36	10.25 ± 2.10	308.29 ± 11.25
FVHB-3	Cangzhou, Hebei	102.81 ± 5.36	31.49 ± 2.88	5.63 ± 1.36	139.94 ± 12.03
FVHB-4	Cangzhou, Hebei	123.15 ± 3.85	42.59 ± 2.56	7.67 ± 1.87	173.41 ± 9.87
FVHB-5	Yongnian, Hebei	11.25 ± 1.39	27.95 ± 3.11	15.65 ± 2.58	54.85 ± 5.36
FVHB-6	Handan, Hebei	5.81 ± 0.97	14.90 ± 1.01	9.10 ± 1.30	29.81 ± 3.99
FVHB-7	Qinhuangdao, Hebei	405.76 ± 11.21	36.85 ± 1.23	6.71 ± 0.56	449.33 ± 8.23
FVHB-8	Luanxian, Hebei	374.05 ± 8.89	32.78 ± 2.58	8.73 ± 0.55	415.55 ± 14.25
FVHB-9	Zhangjiakou, Hebei	62.43 ± 3.25	23.44 ± 0.97	4.98 ± 0.87	90.85 ± 6.86
FVHN-1	Zhengzhou, Henan	54.53 ± 2.55	0.00	3.47 ± 0.64	57.99 ± 7.11
FVHN-2	Zhengzhou, Henan	350.58 ± 9.36	65.81 ± 3.12	8.00 ± 1.02	424.39 ± 13.58
FVHN-3	Zhongmo, Henan	1072.13 ± 23.56	61.19 ± 2.58	42.43 ± 2.37	1175.75 ± 35.56
FVHN-4	Xunxian, Henan	7.46 ± 0.91	8.03 ± 0.46	0.28 ± 0.04	15.77 ± 1.91
FVHN-6	Luoyang, Henan	3195.88 ± 33.51	755.84 ± 6.35	56.30 ± 3.25	4008.02 ± 80.12
FVHN-7	Luoyang, Henan	2886.75 ± 30.12	661.72 ± 5.01	50.12 ± 3.68	3598.59 ± 53.13
FVHN-8	Fangcheng, Henan	280.79 ± 8.87	51.09 ± 1.25	5.83 ± 0.65	337.70 ± 23.14
FVHN-9	Luyi, Henan	276.79 ± 6.84	98.93 ± 2.11	3.85 ± 0.27	379.56 ± 11.08
FVHN-10	Zhumadian, Henan	13,456.63 ± 89.23	1864.90 ± 20.14	96.37 ± 3.87	15,417.91 ± 200.73
FVHLJ-1	Qiqihar, Heilongjiang	2.98 ± 0.31	8.65 ± 0.63	2.69 ± 0.29	14.32 ± 0.86
FVHLJ-2	Qiqihar, Heilongjiang	3.79 ± 0.41	10.25 ± 0.87	3.86 ± 0.27	17.90 ± 0.65
FVHLJ-3	Harbin, Heilongjiang	992.17 ± 8.11	120.22 ± 10.54	18.24 ± 3.45	1130.63 ± 21.21
FVHLJ-4	Harbin, Heilongjiang	3.62 ± 0.65	9.54 ± 0.68	6.16 ± 0.67	19.33 ± 1.22
FVHLJ-5	Suihua, Heilongjiang	5140.81 ± 32.23	652.95 ± 9.35	50.84 ± 2.35	5844.59 ± 23.56
FVHUB-1	Enshi, Hubei	21.54 ± 1.52	8.23 ± 0.21	16.87 ± 3.45	46.64 ± 2.24
FVHUN-2	Changsha, Hunan	51.04 ± 1.88	12.05 ± 0.66	101.46 ± 12.03	164.56 ± 3.21
FVJL-1	Dehui, Jining	18,416.44 ± 66.12	441.6818.11	356.31 ± 15.22	19,214.44 ± 159.32
FVLN-1	Youyan, Liaoning	5.75 ± 0.89	11.38 ± 1.02	14.93 ± 1.28	32.05 ± 2.14
FVLN-2	Donggang, Liaoning	0.00	0.00	0.00	0.00
FVLN-3	Fengcheng, Liaoning	1243.47 ± 13.56	203.89 ± 11.25	66.77 ± 0.89	1514.13 ± 23.14
FVLN-4	Fengcheng, Liaoning	1103.47 ± 11.33	198.35 ± 9.12	53.07 ± 0.45	1354.89 ± 19.88
FVLN-5	Fumeng, Liaoning	281.38 ± 5.78	50.88 ± 3.11	150.28 ± 10.22	482.54 ± 11.25
FVLN-6	Fumeng, Liaoning	18.47 ± 1.23	7.81 ± 0.78	14.35 ± 1.78	40.62 ± 3.32
FVLN-7	Fumeng, Liaoning	3.84 ± 0.75	9.33 ± 0.56	4.39 ± 0.56	17.56 ± 2.11
FVNMG-1	Chifeng, Inner Mongolia	586.63 ± 8.22	245.79 ± 11.23	55.16 ± 4.23	887.58 ± 12.01
FVNMG-2	Wuhai, Inner Mongolia	45.01 ± 3.11	9.25 ± 0.88	44.30 ± 3.55	98.56 ± 12.37
FVNX-1	Tongxin, Ningxia	11.74 ± 1.03	8.67 ± 0.97	9.34 ± 1.02	29.75 ± 3.21
FVNX-2	Yinchuan, Ningxia	213.72 ± 8.47	0.00	8.69 ± 0.89	222.41 ± 22.17
FVNX-3	Yongning, Ningxia	180.78 ± 3.66	39.89 ± 3.54	15.02 ± 1.58	235.68 ± 6.89
FVSD-1	Dezhou, Shandong	0.00	0.00	0.00	0.00
FVSD-2	Dezhou, Shandong	7.78 ± 1.12	6.60 ± 0.65	9.30 ± 1.07	23.68 ± 3.58
FVSD-3	Yuncheng, Shandong	7.91 ± 0.76	5.55 ± 0.77	13.32 ± 0.56	26.77 ± 1.25
FVSD-4	Jiaxiang, Shandong	3.55 ± 0.43	3.40 ± 0.61	1.04 ± 0.11	7.99 ± 0.81
FVSD-5	Jining, Shandong	3.24 ± 0.77	5.39 ± 0.56	3.88 ± 0.57	12.51 ± 0.56
FVSD-6	Jining, Shandong	5.35 ± 0.87	7.69 ± 0.86	5.93 ± 0.64	18.97 ± 0.47
FVSD-7	Sishui, Shandong	88.43 ± 5.44	13.57 ± 1.57	61.29 ± 1.26	163.29 ± 23.57
FVSD-8	Weishan, Shandong	4.14 ± 0.39	3.36 ± 0.67	5.26 ± 0.88	12.77 ± 1.87
FVSD-9	Yanzhou, Shandong	2.52 ± 0.57	3.00 ± 0.25	3.24 ± 0.54	8.75 ± 0.65
FVSD-10	Liaocheng, Shandong	204.66 ± 10.13	61.96 ± 3.31	11.95 ± 1.06	278.58 ± 12.37
FVSD-11	Pingdu, Shandong	2.61 ± 0.13	402.81 ± 23.14	89.43 ± 5.33	494.85 ± 23.11
FVSD-12	Qingzhou, Shandong	1196.33 ± 22.11	73.85 ± 11.01	321.18 ± 11.23	1591.37 ± 20.87
FVSD-13	Shouguang, Shandong,	2405.54 ± 28.32	106.06 ± 5.23	1510.54 ± 46.31	4022.15 ± 42.56
FVSD-14	Laizhou, Shandong	145.34 ± 8.46	25.82 ± 2.45	74.04 ± 8.12	245.20 ± 15.21
FVS-1	Changzhi, Shanxi	4402.45 ± 25.32	555.28 ± 35.43	1123.88 ± 23.21	6081.61 ± 53.25
FVSX-1	Baoji, Shaanxi	312.24 ± 12.78	18.66 ± 2.55	21.95 ± 3.01	352.85 ± 10.04
FVSX-2	Qishan, Shaanxi	253.62 ± 13.33	0.00	13.25 ± 2.06	266.87 ± 16.23
FVSX-3	Danfeng, Shaanxi	4384.86 ± 35.41	287.50 ± 5.37	61.46 ± 3.02	4733.82 ± 35.65
FVSX-4	Shangluo, Shaanxi	3476.72 ± 30.12	351.97 ± 15.36	95.03 ± 3.11	3923.72 ± 40.24
FVSX-5	Shangluo, Shaanxi	2486.72 ± 15.32	231.51 ± 9.31	65.13 ± 2.18	2783.36 ± 29.81
FVSX-6	Luonan, Shaanxi	21.52 ± 1.13	12.06 ± 2.14	16.42 ± 1.56	50.00 ± 3.60
FVSX-7	Luonan, Shaanxi	2383.63 ± 23.08	155.53 ± 6.87	310.49 ± 9.23	2849.66 ± 50.17
FVSX-8	Fuping, Shaanxi	210.44 ± 5.63	56.57 ± 3.47	17.26 ± 1.25	284.27 ± 23.14
FVSX-9	Huxian, Shaanxi	23.54 ± 2.13	0.00	0.00	23.54 ± 2.38
FVSC-1	Zhongjiang, Sichuan	223.58 ± 15.26	0.00	0.00	223.58 ± 10.25
FVSC-2	Santai, Sichuan	2.26 ± 0.23	3.63 ± 0.58	0.88 ± 0.05	6.77 ± 0.67
FVSC-3	Mianyang, Sichuan	77.40 ± 2.36	0.00	0.00	77.40 ± 3.54
FVSC-4	Xichong, Sichuan	17.73 ± 1.09	5.14 ± 0.82	28.86 ± 5.23	51.72 ± 3.14
FVYN-1	Mangshi, Yunnan	72.25 ± 2.11	15.88 ± 1.82	111.17 ± 4.77	199.30 ± 8.12
FVYN-2	Dehong, Yunnan	0.00	0.00	0.00	0.00
FVYN-3	Mile, Yunnan	40.23 ± 5.21	0.00	0.00	40.23 ± 3.69
FVYN-4	Qujing, Yunnan	114.54 ± 5.67	0.00	10.07 ± 0.78	124.61 ± 10.25

**Table 2:** Mycotoxin chemotype and production of *Fusarium graminearum* species complex isolates.

No.	Species	Isolate of Origin	Chemotype	DON (µg/g)	15-ADON (µg/g)	3-ADON (µg/g)	ZEN (µg/g)
FG001	*F. g.*	Mengcheng, Anhui	15-ADON	7392.35 ± 48.02	27,713.62 ± 181.02	8316.53 ± 86.32	0.00
FG008	*F. g.*	Shunyi, Beijing	15-ADON	304.06 ± 15.36	1920.07 ± 23.36	528.73 ± 23.21	46.31 ± 3.25
FG012	*F. g.*	Zhuanglang, Gansu	15-ADON	19,795.33 ± 99.23	4350.49 ± 35.14	1315.46 ± 35.25	6.95 ± 0.81
FG023	*F. g.*	Changli, Hebei	15-ADON	4319.20 ± 35.62	24,411.32 ± 150.12	8167.15 ± 78.23	0.00
FG029	*F. g.*	Zhangjiakou, Hebei	15-ADON	12,464.99 ± 88.25	33,162.16 ± 223.57	11,471.99 ± 113.14	198.96 ± 13.37
FG030	*F. g.*	Tangshan, Hebei	15-ADON	3898.09 ± 46.35	18,546.56 ± 88.17	5674.04 ± 53.62	0.00
FG039	*F. g.*	Xiangcheng, Henan	15-ADON	0.00	0.00	0.00	0.00
FG042	*F. g.*	Sanmenxia, Henan	15-ADON	3189.00 ± 23.56	18,175.55 ± 100.25	5297.00 ± 85.36	0.00
FG043	*F. g.*	Zhoukou, Henan	15-ADON	394.64 ± 12.23	5956.24 ± 65.36	1863.57 ± 36.52	0.00
FG044	*F. g.*	Zhumadian, Henan	15-ADON	7955.22 ± 65.57	34,732.64 ± 137.58	9981.63 ± 89.65	0.00
FG050	*F. g.*	Qiqihar, Heilongjiang	15-ADON	4267.29 ± 23.39	6453.04 ± 82.21	1797.64 ± 13.56	0.00
FG060	*F. g.*	Donggang, Liaoning	15-ADON	406.97 ± 12.35	6967.08 ± 56.34	1718.66 ± 56.23	0.00
FG063	*F. g.*	Shenyang, Liaoning	15-ADON	1757.88 ± 21.45	4615.86 ± 58.75	1328.25 ± 33.21	2.73 ± 0.51
FG072	*F. g.*	Pingdu, Shandong	15-ADON	3172.77 ± 39.23	12,843.81 ± 98.21	3430.94 ± 25.24	6.18 ± 0.39
FG075	*F. g.*	Jiaxiang, Shandong	15-ADON	685.04 ± 15.63	10,420.73 ± 89.45	2869.32 ± 33.21	55.57 ± 3.21
FG078	*F. g.*	Dezhou, Shandong	15-ADON	8566.56 ± 46.36	54,382.05 ± 256.32	17,855.15 ± 78.56	6.83 ± 1.02
FG080	*F. g.*	Liaocheng, Shandong	15-ADON	224.59 ± 22.01	857.22 ± 10.58	242.27 ± 11.24	0.00
FG081	*F. g.*	Liaocheng, Shandong	15-ADON	1308.70 ± 63.70	9308.46 ± 56.32	2934.81 ± 55.21	22.72 ± 0.97
FG082	*F. g.*	Tai`an, Shandong	15-ADON	0.00	72.99 ± 6.38	23.20 ± 2.12	0.00
FG083	*F. g.*	Yanzhou, Shandong	15-ADON	213.08 ± 13.54	1704.10 ± 20.12	451.66 ± 10.53	0.00
FG084	*F. g.*	Qingdao, Shandong	15-ADON	0.00	25.52 ± 2.58	6.04 ± 0.65	0.00
FG086	*F. g.*	Laizhou, Shandong	15-ADON	1419.21 ± 21.08	7742.05 ± 33.56	1956.23 ± 21.32	0.00
FG087	*F. g.*	Weishan, Shandong	15-ADON	2417.01 ± 22.01	11,843.94 ± 87.58	2677.59 ± 35.26	0.00
FG096	*F. g.*	Xianyang, Shaanxi	15-ADON	1201.27 ± 36.25	8910.50 ± 98.24	2572.49 ± 63.27	0.00
FG098	*F. g.*	Fengxiang, Shaanxi	15-ADON	13.35 ± 1.04	86.84 ± 5.63	9.51 ± 0.59	0.00
FG099	*F. g.*	Yulin, Shaanxi	15-ADON	0.00	47.01 ± 6.23	10.14 ± 0.26	0.00
FG101	*F. g.*	Shangnan, Shaanxi	15-ADON	4764.23 ± 55.36	20,461.49 ± 100.58	5694.38 ± 66.39	0.00
FG103	*F. g.*	Zhashui, Shaanxi	15-ADON	2710.03 ± 12.37	8006.75 ± 89.57	2286.71 ± 55.31	0.00
FG135	*F. g.*	Sanmenxia, Henan	15-ADON	866.30 ± 26.31	4106.67 ± 76.35	1164.43 ± 53.37	1.77 ± 0.55
FG141	*F. g.*	Heishan, Liaoning	15-ADON	1668.64 ± 33.10	9666.53 ± 100.57	2425.36 ± 51.98	0
FG144	*F. g.*	Xianfeng, Hubei	15-ADON	5915.73 ± 29.25	26,784.49 ± 85.69	6497.77 ± 88.56	0.00
FG144	*F. g.*	Heihe, Helongjiang	15-ADON	0	174.24 ± 12.35	27.75 ± 3.04	3.63 ± 0.34
FG147	*F. g.*	Wangkui, Helongjiang	15-ADON	170.41 ± 8.63	311.93 ± 23.21	69.48 ± 3.56	88.27 ± 2.78
FG150	*F. g.*	Jiamusi, Helongjiang	15-ADON	12,422.55 ± 119.65	16,823.61 ± 88.56	4803.78 ± 40.12	0
FG153	*F. g.*	Fujin, Helongjiang	15-ADON	4742.29 ± 63.21	23,219.59 ± 150.69	6518.67 ± 45.23	0
FG155	*F. g.*	Fujin, Helongjiang	15-ADON	1825.81 ± 25.78	8084.01 ± 80.23	2064.13 ± 35.55	0
FG156	*F. g.*	Fengcheng, Liaoning	15-ADON	507.41 ± 13.69	2925.37 ± 23.01	575.05 ± 10.27	0
FG158	*F. g.*	Panshi, Jilin	15-ADON	16,723.72 ± 87.56	81,539.49 ± 300.57	19,590.61 ± 211.25	0
FG159	*F. g.*	Panshi, Jilin	15-ADON	698.51 ± 4.68	2845.5 ± 23.45	887.61 ± 6.38	0
FG162	*F. g.*	Nenjiang, Helongjiang	15-ADON	929.66 ± 15.23	5251.46 ± 23.22	1298.79 ± 37.24	14.37 ± 1.39
FG165	*F. g.*	Qinggang, Helongjiang	15-ADON	2998.93 ± 23.24	23,152.65 ± 123.5	5391.17 ± 52.13	430.24 ± 25.87
FG015	*F. m.*	Puding, Guizhou	NIV	0.00	5.43 ± 0.56	17.24 ± 1.66	0.00
FG017	*F. m.*	Bijie, Guizhou	NIV	0.00	0.00	0.00	0.00
FG019	*F. m.*	Guiyang, Guizhou	NIV	0.00	20.46 ± 1.23	470.83 ± 12.25	0.00
FG020	*F. m.*	Zunyi, Guizhou	NIV	0.00	28.82 ± 1.21	151.29 ± 13.24	0.00
FG100	*F. m.*	Luonan, Shaanxi	NIV	0.00	67.44 ± 2.25	709.58 ± 33.22	0.00
FG102	*F. m.*	Danfeng, Shaanxi	15-ADON, NIV	0.00	12.29 ± 2.58	13.85 ± 1.65	0.00
FG104	*F. m.*	Zhenan, Shaanxi	15-ADON	69.60 ± 6.87	222.33 ± 11.012	63.93 ± 3.77	0.00
FG105	*F. m.*	Shangluo, Shaanxi	15-ADON, NIV	0.00	47.25 ± 3.12	53.13 ± 5.20	0.00
FG106	*F. m.*	Huayin, Shaanxi	NIV	0.00	55.75 ± 1.25	767.60 ± 45.31	2.11 ± 0.49
FG107	*F. m.*	Luonan, Shaanxi	NIV	0.00	40.05 ± 3.24	0.00	0.00
FG128	*F. m.*	Qujing, Yunnan	NIV	0.00	8.58 ± 0.54	0.00	0.00
FG129	*F. m.*	Qujing, Yunnan	NIV	0.00	11.46 ± 0.89	284.86 ± 23.56	8.90 ± 0.87
FG130	*F. m.*	Mile, Yunnan	NIV	0.00	14.24 ± 1.55	78.62 ± 9.39	0.00
FG133	*F. m.*	Luxi, Yunnan	NIV	0.00	104.90 ± 9.25	3519.87 ± 33.21	0.00
FG140	*F. m.*	Yuxi, Yunnan	NIV	0.00	0.00	0.00	0.00
FG142	*F. m.*	Badong, Hubei	NIV	0.00	0.00	0.00	0.00
FG143	*F. m.*	Jianshi, Hubei	NIV	0.00	0.00	0.00	0.00
FG145	*F. m.*	Xianfeng, Hubei	NIV	0.00	0.00	0.00	0.00
FG146	*F. m.*	Pixian, Sichuan	NIV	0.00	0.00	0.00	0.00
FG151	*F. m.*	Mianyang, Sichuan	15-ADON	0.00	0.00	0.00	0.00
FG152	*F. m.*	Jianyang, Sichuan	15-ADON	0.00	0.00	0.00	0.00
FG010	*F. b.*	Haidian, Beijng	15-ADON	371.46 ± 12.56	2876.36 ± 36.56	843.70 ± 23.10	10.64 ± 1.11
FG028	*F. b.*	Zhangjiakou, Hebei	15-ADON	3636.37 ± 46.38	13,845.04 ± 95.63	4475.88 ± 45.32	0.00
FG057	*F. b.*	Huinan, Jilin	15-ADON	6794.02 ± 45.57	6844.28 ± 98.76	2132.45 ± 33.12	17.39 ± 0.97
FG069	*F. b.*	Chifeng, Inner Mongolia	15-ADON	9439.56 ± 80.67	9901.51 ± 85.69	2795.33 ± 46.29	0.00
FG092	*F. b.*	Changzhi, Shanxi	15-ADON	5629.31 ± 36.87	5687.94 ± 36.54	1779.75 ± 30.01	53.51 ± 3.24
FG094	*F. b.*	Changzhi, Shanxi	15-ADON	9939.69 ± 55.36	11,725.34 ± 111.30	3457.11 ± 55.32	0.00
FG142	*F. b.*	Suihua, Helongjiang	15-ADON	1491.91 ± 23.25	4549.23 ± 56.68	1334.72 ± 23.65	14.25 ± 2.16
FG145	*F. b.*	Tongliao, Inner Mongolia	15-ADON	3151.85 ± 53.23	7899.02 ± 36.89	2168.33 ± 33.45	0
FG146	*F. b.*	Tongliao, Inner Mongolia	15-ADON	0	38.83 ± 3.87	0	0
FG147	*F. b.*	Chengdu, Sichuan	15-ADON	0.00	0.00	0.00	0.00
FG148	*F. b.*	Liangshan, Sichuan	15-ADON	1156.47 ± 23.15	22123.45 ± 78.65	5838.04 ± 39.87	0.00
FG149	*F. b.*	Qiqihar, Helongjiang	15-ADON	1548.25 ± 35.24	6991.64 ± 36.56	2114.88 ± 24.21	60.05 ± 3.25
FG160	*F. b.*	Lanxi, Helongjiang	15-ADON	4236.92 ± 23.47	18455.36 ± 100.24	4741.94 ± 55.12	36.92 ± 3.67
FG161	*F. b.*	Qinggang, Helongjiang	15-ADON	518.48 ± 12.25	5451.17 ± 85.63	1408.57 ± 23.23	74.67 ± 5.26
FG166	*F. b.*	Zhenlai, Jilin	15-ADON	1496.17 ± 22.01	5324.07 ± 83.21	1386.40 ± 23.87	22.68 ± 2.98
FG167	*F. b.*	Huachuan, Helongjiang	15-ADON	14.20 ± 1.25	236.46 ± 23.56	153.75 ± 9.68	29.61 ± 2.65

Key: *F. g.*, *Fusarium graminearum*; *F. m.*, *Fusarium meridionale*; *F. b.*, *Fusarium boothii*.

**Table 3:** Distribution of 239 affected maize ear and seed samples.

Province	Number of Counties	Province	Number of Counties
Anhui	5	Inner mongoria	3
Beijing	2	Jilin	4
Gansu	2	Liaoning	5
Guizhou	6	Ningxia	3
Hebei	12	Shaanxi	14
Heilongjiang	8	Shandong	13
Henan	11	Shanxi	4
Hubei	4	Sichuan	7
Hunan	1	Yunnan	6

**Table 4:** Specific primers for *Fusarium* spp. used in this study.

Fungus	Primer	Sequence (5′–3′)	Target Fragment (bp)	Tm (°C)	Reference
*Fusarium* spp.	ItsF	AACTCCCAAACCCCTGTGAACATA	431	58	[49]
	ItsR	TTTAACGGCGTGGCCGC			
*F. graminearum*	Fg16NF	ACAGATGACAAGATTCAGGCACA	280	57	[50]
	Fg16NR	TTCTTTGACATCTGTTCAACCCA			
*F. culmorum*	Fc01F	ATGGTGAACTCGTCCTGGC	570	59	[50]
	Fc01R	CCCTTCTTACGCCAATCTCG			
*F. oxysporum*	FoF1	ACATACCACTTGTTGCCTCG	340	58	[51]
	FoR1	CGCCAATCAATTTGAGGAACG			
*F. verticillioides*	VER1	CTTCCTGCGATGTTTCTCC	578	56	[52]
	VER2	AATTGGCCATTGGTATTATATATCTA			
*F. proliferatum*	PRO1	CTTTCCGCCAAGTTTCTTC	585	56	[52]
	PRO2	TGTCAGTAACTCGACGTTGTTG			
*F. subglutinans*	SUB1	CTGTCGCTAACCTCTTTATCCA	631	56	[52]
	SUB2	CAGTATGGACGTTGGTATTATATCTAA			

**Table 5:** The information of three standard reference strains in this study.

Taxon	Collection Strain No.	Accession No.	Host	Geographic Origin
*F. graminearum*	VI 01028	AJ543588.1	Oat	Norway
*F. meridionale*	28723	AF212436.1	Corn	Nepal
*F. boothii*	26916	AF212444.1	Corn	South Africa

## Supplementary Materials

The following are available online at www.mdpi.com/2072-6651/8/6/186/s1, Figure S1: The alignments of *TEF-1*α gene sequences of FGSC isolates and standard reference strains.

## Abbreviations

The following abbreviations are used in this manuscript:

3-ADON	3-acetyl-deoxynivalenol
15-ADON	15-deoxynivalenol
DON	deoxynivalenol
FB	fumonisin B
FGSC	*Fusarium graminearum* species complex
HPLC	high-performance liquid chromatography
LC-MS/MS	liquid chromatography-tandem mass spectrometry
ML	maximum likelihood
NIV	nivalenol
OPA	o-phthaldialdehyde
PCR	polymerase chain reactions
PDA	potato dextrose agar
SNA	Spezieller Nährstoffarmer agar
*TEF*	translation elongation factor
UHPLC-MS/MS	ultra-high performance liquid chromatography-mass spectrometry
ZEN	zearalenone
