# Supplementary material for: Identification of Pathogenic Fusarium spp. Causing Maize Ear Rot and Potential Mycotoxin Production in China
Source: Toxins (Basel). 2016 Jun 21;8(6):186. doi: 10.3390/toxins8060186 (PMC4926152; doi:10.3390/toxins8060186)
Supplement: Supplementary file 1 [file toxins-08-00186-s001.pdf]

Toxins **2016**, *8*, 186; doi:10.3390/toxins8060186

Supplementary Materials: Identification of Pathogenic *Fusarium* spp. Causing Maize Ear Rot and Poten tial Mycotoxin Production in China

Canxing Duan, Zihui Qin, Zhihuan Yang, Weixi Li, Suli Sun, Zhendong Zhu and Xiaoming Wang

```
AF212436.1_Fusar                         TCGTCGTCATCGGCCACGTCGACTCTGGCAAGTCGACCACTG     42
AF212444.1_Fusar                         TCGTCGTCATCGGCCACGTCGACTCTGGCAAGTCGACCACTG     42
AJ543588.1_Fusar                         TCGTCGTCATCGGCCACGTCGACTCTGGCAAGTCGACCACTG     42
FG-010               GGACAAGACTCACCTTAACGTCGTCGTCATCGGCCACGTCGACTCTGGCAAGTCGACCACTG     62
FG-015                GACAAGACTCACCTTAACGTCGTCGTCATCGGCCACGTCGACTCTGGCAAGTCGACCACTG     61
FG-017                                     GTCGTCATCGGCCACGTCGACTCTGGCAAGTCGACCACTG     40
FG-019                   CAAGACTCACCTTAACGTCGTCGTCATCGGCCACGTCGACTCTGGCAAGTCGACCACTG     59
FG-020                    AAGACTCACCTTAACGTCGTCGTCATCGGCCACGTCGACTCTGGCAAGTCGACCACTG     58
FG-029                    AAGACTCACCTTAACGTCGTCGTCATCGGCCACGTCGACTCTGGCAAGTCGACCACTG     58
FG-030                    AAGACTCACCTTAACGTCGTCGTCATCGGCCACGTCGACTCTGGCAAGTCGACCACTG     58
FG-039              AGGACAAGACTCACCTTAACGTCGTCGTCATCGGCCACGTCGACTCTGGCAAGTCGACCACTG     63
FG-043                      GACTCACCTTAACGTCGTCGTCATCGGCCACGTCGACTCTGGCAAGTCGACCACTG     56
FG-044                     AGACTCACCTTAACGTCGTCGTCATCGGCCACGTCGACTCTGGCAAGTCGACCACTG     57
FG-057                   CAAGACTCACCTTAACGTCGTCGTCATCGGCCACGTCGACTCTGGCAAGTCGACCACTG     59
FG-078                   CAAGACTCACCTTAACGTCGTCGTCATCGGCCACGTCGACTCTGGCAAGTCGACCACTG     59
FG-080                    AAGACTCACCTTAACGTCGTCGTCATCGGCCACGTCGACTCTGGCAAGTCGACCACTG     58
FG-082              AGGACAAGACTCACCTTAACGTCGTCGTCATCGGCCACGTCGACTCTGGCAAGTCGACCACTG     63
FG-086                    AAGACTCACCTTAACGTCGTCGTCATCGGCCACGTCGACTCTGGCAAGTCGACCACTG     58
FG-087                     AGACTCACCTTAACGTCGTCGTCATCGGCCACGTCGACTCTGGCAAGTCGACCACTG     57
FG-092                    AAGACTCACCTTAACGTCGTCGTCATCGGCCACGTCGACTCTGGCAAGTCGACCACTG     58
FG-094                     AGACTCACCTTAACGTCGTCGTCATCGGCCACGTCGACTCTGGCAAGTCGACCACTG     57
FG-096              AGGACAAGACTCACCTTAACGTCGTCGTCATCGGCCACGTCGACTCTGGCAAGTCGACCACTG     63
FG-098                    AAGACTCACCTTAACGTCGTCGTCATCGGCCACGTCGACTCTGGCAAGTCGACCACTG     58
FG-100                    AAGACTCACCTTAACGTCGTCGTCATCGGCCACGTCGACTCTGGCAAGTCGACCACTG     58
FG-102                   CAAGACTCACCTTAACGTCGTCGTCATCGGCCACGTCGACTCTGGCAAGTCGACCACTG     59
FG-103                   CAAGACTCACCTTAACGTCGTCGTCATCGGCCACGTCGACTCTGGCAAGTCGACCACTG     59
FG-104                                         TCATCGCCCACGTCGACTCTGGCAAGTCGACCACTG     36
FG-106              AGGACAAGACTCACCTTAACGTCGTCGTCATCGGCCACGTCGACTCTGGCAAGTCGACCACTG     63
FG-107               GGACAAGACTCACCTTAACGTCGTCGTCATCGGCCACGTCGACTCTGGCAAGTCGACCACTG     62
FG-128                                                                  AGTCGACCACTG     12
FG-129               GGACAAGACTCACCTTAACGTCGTCGTCATCGGCCACGTCGACTCTGGCAAGTCGACCACTG     62
FG-130                    AAGACTCACCTTAACGTCGTCGTCATCGGCCACGTCGACTCTGGCAAGTCGACCACTG     58
FG-135                   CAAGACTCACCTTAACGTCGTCGTCATCGGCCACGTCGACTCTGGCAAGTCGACCACTG     59

AF212436.1_Fusar    TGAGTACCACCACATCCAACCCCGCCGACACTTGGCGGGG.TAGTTTCAAATTTCCAACGTG    104
AF212444.1_Fusar    TGAGTACCACCGCATCCAACCCCGCCGACACTTGGCGGGGGTAGTTTCAAATTTCCAATGTG    105
AJ543588.1_Fusar    TGAGTACCACCGCATCCAACCCCGCCGACACTTGGCGGGG.TAGTTTCAAATTTCCAATGTG    104
FG-010              TGAGTACCACCGCATCCAACCCCGCCGACACTTGGCGGGGGTAGTTTCAAATTTCCAATGTG    125
FG-015              TGAGTACCACCACATCCAACCCCGCCGACACTTGGCGGGG.TAGTTTCAAATTTCCAACGTG    123
FG-017              TGAGTACCACCACATCCAACCCCGCCGACACTTGGCGGGG.TAGTTTCAAATTTCCAACGTG    102
FG-019              TGAGTACCACCACATCCAACCCCGCCGACACTTGGCGGGG.TAGTTTCAAATTTCCAACGTG    121
FG-020              TGAGTACCACCACATCCAACCCCGCCGACACTTGGCGGGG.TAGTTTCAAATTTCCAACGTG    120
FG-029              TGAGTACCACCGCATCCAACCCCGCCGACACTTGGCGGGG.TAGTTTCAAATTTCCAATGTG    120
FG-030              TGAGTACCACCGCATCCAACCCCGCCGACACTTGGCGGGG.TAGTTTCAAATTTCCAATGTG    120
FG-039              TGAGTACCACCGCATCCAACCCCGCCGACACTTGGCGGGG.TAGTTTCAAATTTCCAATGTG    125
FG-043              TGAGTACCACCGCATCCAACCCCGCCGACACTTGGCGGGG.TAGTTTCAAATTTCCAATGTG    118
FG-044              TGAGTACCACCGCATCCAACCCCGCCGACACTTGGCGGGG.TAGTTTCAAATTTCCAATGTG    119
FG-057              TGAGTACCACCGCATCCAACCCCGCCGACACTTGGCGGGGGTAGTTTCAAATTTCCAATGTG    122
FG-078              TGAGTACCACCGCATCCAACCCCGCCGACACTTGGCGGGG.TAGTTTCAAATTTCCAATGTG    121
FG-080              TGAGTACCACCGCATCCAACCCCGCCGACACTTGGCGGGG.TAGTTTCAAATTTCCAATGTG    120
FG-082              TGAGTACCACCGCATCCAACCCCGCCGACACTTGGCGGGG.TAGTTTCAAATTTCCAATGTG    125
FG-086              TGAGTACCACCGCATCCAACCCCGCCGACACTTGGCGGGG.TAGTTTCAAATTTCCAATGTG    120
FG-087              TGAGTACCACCGCATCCAACCCCGCCGACACTTGGCGGGG.TAGTTTCAAATTTCCAATGTG    119
FG-092              TGAGTACCACCGCATCCAACCCCGCCGACACTTGGCGGGGGTAGTTTCAAATTTCCAATGTG    121
FG-094              TGAGTACCACCGCATCCAACCCCGCCGACACTTGGCGGGGGTAGTTTCAAATTTCCAATGTG    120
FG-096              TGAGTACCACCGCATCCAACCCCGCCGACACTTGGCGGGG.TAGTTTCAAATTTCCAATGTG    125
FG-098              TGAGTACCACCGCATCCAACCCCGCCGACACTTGGCGGGG.TAGTTTCAAATTTCCAATGTG    120
FG-100              TGAGTACCACCACATCCAACCCCGCCGACACTTGGCGGGG.TAGTTTCAAATTTCCAACGTG    120
FG-102              TGAGTACCACCACATCCAACCCCGCCGACACTTGGCGGGG.TAGTTTCAAATTTCCAACGTG    121
FG-103              TGAGTACCACCGCATCCAACCCCGCCGACACTTGGCGGGG.TAGTTTCAAATTTCCAATGTG    121
FG-104              TGAGTACCACCACATCCAACCCCGCCGACACTTGGCGGGG.TAGTTTCAAATTTCCAACGTG     98
FG-106              TGAGTACCACCACATCCAACCCCGCCGACACTTGGCGGGG.TAGTTTCAAATTTCCAACGTG    125
FG-107              TGAGTACCACCACATCCAACCCCGCCGACACTTGGCGGGG.TAGTTTCAAATTTCCAACGTG    124
FG-128              TGAGTACCACCACATCCAACCCCGCCGACACTTGGCGGGG.TAGTTTCAAATTTCCAACGTG     74
FG-129              TGAGTACCACCACATCCAACCCCGCCGACACTTGGCGGGG.TAGTTTCAAATTTCCAACGTG    124
FG-130              TGAGTACCACCACATCCAACCCCGCCGACACTTGGCGGGG.TAGTTTCAAATTTCCAACGTG    120
FG-135              TGAGTACCACCGCATCCAACCCCGCCGACACTTGGCGGGG.TAGTTTCAAATTTCCAATGTG    121
```

```
AF212436.1_Fusar   CTGACATACTTTGATAGACCGGTCACTTGATCTACCAGTGCGGTGGTATCGACAAGCGAACCA   167
AF212444.1_Fusar   CTGACATACTTTGATAGACCGGTCACTTGATCTACCAGTGCGGTGGTATCGACAAGCGAACCA   168
AJ543588.1_Fusar   CTGACATACTTTGATAGACCGGTCACTTGATCTACCAGTGCGGTGGTATCGACAAGCGAACCA   167
FG-010             CTGACATACTTTGATAGACCGGTCACTTGATCTACCAGTGCGGTGGTATCGACAAGCGAACCA   188
FG-015             CTGACATACTTTGATAGACCGGTCACTTGATCTACCAGTGCGGTGGTATCGACAAGCGAACCA   186
FG-017             CTGACATACTTTGATAGACCGGTCACTTGATCTACCAGTGCGGTGGTATCGACAAGCGAACCA   165
FG-019             CTGACATACTTTGATAGACCGGTCACTTGATCTACCAGTGCGGTGGTATCGACAAGCGAACCA   184
FG-020             CTGACATACTTTGATAGACCGGTCACTTGATCTACCAGTGCGGTGGTATCGACAAGCGAACCA   183
FG-029             CTGACATACTTTGATAGACCGGTCACTTGATCTACCAGTGCGGTGGTATCGACAAGCGAACCA   183
FG-030             CTGACATACTTTGATAGACCGGTCACTTGATCTACCAGTGCGGTGGTATCGACAAGCGAACCA   183
FG-039             CTGACATACTTTGATAGACCGGTCACTTGATCTACCAGTGCGGTGGTATCGACAAGCGAACCA   188
FG-043             CTGACATACTTTGATAGACCGGTCACTTGATCTACCAGTGCGGTGGTATCGACAAGCGAACCA   181
FG-044             CTGACATACTTTGATAGACCGGTCACTTGATCTACCAGTGCGGTGGTATCGACAAGCGAACCA   182
FG-057             CTGACATACTTTGATAGACCGGTCACTTGATCTACCAGTGCGGTGGTATCGACAAGCGAACCA   185
FG-078             CTGACATACTTTGATAGACCGGTCACTTGATCTACCAGTGCGGTGGTATCGACAAGCGAACCA   184
FG-080             CTGACATACTTTGATAGACCGGTCACTTGATCTACCAGTGCGGTGGTATCGACAAGCGAACCA   183
FG-082             CTGACATACTTTGATAGACCGGTCACTTGATCTACCAGTGCGGTGGTATCGACAAGCGAACCA   188
FG-086             CTGACATACTTTGATAGACCGGTCACTTGATCTACCAGTGCGGTGGTATCGACAAGCGAACCA   183
FG-087             CTGACATACTTTGATAGACCGGTCACTTGATCTACCAGTGCGGTGGTATCGACAAGCGAACCA   182
FG-092             CTGACATACTTTGATAGACCGGTCACTTGATCTACCAGTGCGGTGGTATCGACAAGCGAACCA   184
FG-094             CTGACATACTTTGATAGACCGGTCACTTGATCTACCAGTGCGGTGGTATCGACAAGCGAACCA   183
FG-096             CTGACATACTTTGATAGACCGGTCACTTGATCTACCAGTGCGGTGGTATCGACAAGCGAACCA   188
FG-098             CTGACATACTTTGATAGACCGGTCACTTGATCTACCAGTGCGGTGGTATCGACAAGCGAACCA   183
FG-100             CTGACATACTTTGATAGACCGGTCACTTGATCTACCAGTGCGGTGGTATCGACAAGCGAACCA   183
FG-102             CTGACATACTTTGATAGACCGGTCACTTGATCTACCAGTGCGGTGGTATCGACAAGCGAACCA   184
FG-103             CTGACATACTTTGATAGACCGGTCACTTGATCTACCAGTGCGGTGGTATCGACAAGCGAACCA   184
FG-104             CTGACATACTTTGATAGACCGGTCACTTGATCTACCAGTGCGGTGGTATCGACAAGCGAACCA   161
FG-106             CTGACATACTTTGATAGACCGGTCACTTGATCTACCAGTGCGGTGGTATCGACAAGCGAACCA   188
FG-107             CTGACATACTTTGATAGACCGGTCACTTGATCTACCAGTGCGGTGGTATCGACAAGCGAACCA   187
FG-128             CTGACATACTTTGATAGACCGGTCACTTGATCTACCAGTGCGGTGGTATCGACAAGCGAACCA   137
FG-129             CTGACATACTTTGATAGACCGGTCACTTGATCTACCAGTGCGGTGGTATCGACAAGCGAACCA   187
FG-130             CTGACATACTTTGATAGACCGGTCACTTGATCTACCAGTGCGGTGGTATCGACAAGCGAACCA   183
FG-135             CTGACATACTTTGATAGACCGGTCACTTGATCTACCAGTGCGGTGGTATCGACAAGCGAACCA   184

AF212436.1_Fusar   TCGAGAAGTTCGAGAAGGTTGGTCTCATTTTCCTCGATCGCGCGCCTTTTTCCCTTTCGAAAT   230
AF212444.1_Fusar   TCGAGAAGTTCGAGAAGGTTGGTCTCATTTTCCTCGATCGCGCGCCCTTTTCCCTTTCGAAAT   231
AJ543588.1_Fusar   TCGAGAAGTTCGAGAAGGTTGGTCTCATTTTCCTCGATCGCGCGCCCTTTTCCCTTTCGAAAT   230
FG-010             TCGAGAAGTTCGAGAAGGTTGGTCTCATTTTCCTCGATCGCGCGCCCTTTTCCCTTTCGAAAT   251
FG-015             TCGAGAAGTTCGAGAAGGTTGGTCTCATTTTCCTCGATCGCGCGCCTTTTTCCCTTTCGAAAT   249
FG-017             TCGAGAAGTTCGAGAAGGTTGGTCTCATTTTCCTCGATCGCGCGCCTTTTTCCCTTTCGAAAT   228
FG-019             TCGAGAAGTTCGAGAAGGTTGGTCTCATTTTCCTCGATCGCGCGCCTTTTTCCCTTTCGAAAT   247
FG-020             TCGAGAAGTTCGAGAAGGTTGGTCTCATTTTCCTCGATCGCGCGCCTTTTTCCCTTTCGAAAT   246
FG-029             TCGAGAAGTTCGAGAAGGTTGGTCTCATTTTCCTCGATCGCGCGCCCTTT.CCCTTTCGAAAT   245
FG-030             TCGAGAAGTTCGAGAAGGTTGGTCTCATTTTCCTCGATCGCGCGCCCTTT.CCCTTTCGAAAT   245
FG-039             TCGAGAAGTTCGAGAAGGTTGGTCTCATTTTCCTCGATCGCGCGCCCTTTTCCCTTTCGAAAT   251
FG-043             TCGAGAAGTTCGAGAAGGTTGGTCTCATTTTCCTCGATCGCGCGCCCTTTTCCCTTTCGAAAT   244
FG-044             TCGAGAAGTTCGAGAAGGTTGGTCTCATTTTCCTCGATCGCGCGCCCTTT.CCCTTTCGAAAT   244
FG-057             TCGAGAAGTTCGAGAAGGTTGGTCTCATTTTCCTCGATCGCGCGCCCTTTTCCCTTTCGAAAT   248
FG-078             TCGAGAAGTTCGAGAAGGTTGGTCTCATTTTCCTCGATCGCGCGCCCTTT.CCCTTTCGAAAT   246
FG-080             TCGAGAAGTTCGAGAAGGTTGGTCTCATTTTCCTCGATCGCGCGCCCTTT.CCCTTTCGAAAT   245
FG-082             TCGAGAAGTTCGAGAAGGTTGGTCTCATTTTCCTCGATCGCGCGCCCTTT.CCCTTTCGAAAT   250
FG-086             TCGAGAAGTTCGAGAAGGTTGGTCTCATTTTCCTCGATCGCGCGCCCTTTTCCCTTTCGAAAT   246
FG-087             TCGAGAAGTTCGAGAAGGTTGGTCTCATTTTCCTCGATCGCGCGCCCTTT.CCCTTTCGAAAT   244
FG-092             TCGAGAAGTTCGAGAAGGTTGGTCTCATTTTCCTCGATCGCGCGCCCTTTTCCCTTTCGAAAT   247
FG-094             TCGAGAAGTTCGAGAAGGTTGGTCTCATTTTCCTCGATCGCGCGCCCTTTTCCCTTTCGAAAT   246
FG-096             TCGAGAAGTTCGAGAAGGTTGGTCTCATTTTCCTCGATCGCGCGCCCTTT.CCCTTTCGAAAT   250
FG-098             TCGAGAAGTTCGAGAAGGTTGGTCTCATTTTCCTCGATCGCGCGCCCTTT.CCCTTTCGAAAT   245
FG-100             TCGAGAAGTTCGAGAAGGTTGGTCTCATTTTCCTCGATCGCGCGCCTTTTTCCCTTTCGAAAT   246
FG-102             TCGAGAAGTTCGAGAAGGTTGGTCTCATTTTCCTCGATCGCGCGCCTTTTTCCCTTTCGAAAT   247
FG-103             TCGAGAAGTTCGAGAAGGTTGGTCTCATTTTCCTCGATCGCGCGCCCTTTTCCCTTTCGAAAT   247
FG-104             TCGAGAAGTTCGAGAAGGTTGGTCTCATTTTCCTCGATCGCGCGCCTTTTTCCCTTTCGAAAT   224
FG-106             TCGAGAAGTTCGAGAAGGTTGGTCTCATTTTCCTCGATCGCGCGCCTTTTTCCCTTTCGAAAT   251
FG-107             TCGAGAAGTTCGAGAAGGTTGGTCTCATTTTCCTCGATCGCGCGCCTTTTTCCCTTTCGAAAT   250
FG-128             TCGAGAAGTTCGAGAAGGTTGGTCTCATTTTCCTCGATCGCGCGCCTTTTTCCCTTTCGAAAT   200
FG-129             TCGAGAAGTTCGAGAAGGTTGGTCTCATTTTCCTCGATCGCGCGCCTTTTTCCCTTTCGAAAT   250
FG-130             TCGAGAAGTTCGAGAAGGTTGGTCTCATTTTCCTCGATCGCGCGCCTTTTTCCCTTTCGAAAT   246
FG-135             TCGAGAAGTTCGAGAAGGTTGGTCTCATTTTCCTCGATCGCGCGCCCTTTTCCCTTTCGAAAT   247
```

```
AF212436.1_Fusar    ATCATTCGAATCGCCCTCACACGACGACTCGATACGCGCCTGTTACCCCGCTCGAGGTCAAAA    293
AF212444.1_Fusar    ATCATTCGAATCGCCCTCACACGACGACTCGATACGCGCCTGTTACCCCGCTCGAGGTCAAAA    294
AJ543588.1_Fusar    ATCATTCGAATCGCCCTCACACGACGACTCGATACGCGCCTGTTACCCCGCTCGAGGTCAAAA    293
FG-010              ATCATTCGAATCGCCCTCACACGACGACTCGATACGCGCCTGTTACCCCGCTCGAGGTCAAAA    314
FG-015              ATCATTCGAATCGCCCTCACACGACGACTCGATACGCGCCTGTTACCCCGCTCGAGGTCAAAA    312
FG-017              ATCATTCGAATCGCCCTCACACGACGACTCGATACGCGCCTGTTACCCCGCTCGAGGTCAAAA    291
FG-019              ATCATTCGAATCGCCCTCACACGACGACTCGATACGCGCCTGTTACCCCGCTCGAGGTCAAAA    310
FG-020              ATCATTCGAATCGCCCTCACACGACGACTCGATACGCGCCTGTTACCCCGCTCGAGGTCAAAA    309
FG-029              ATCATTCGAATCGCCCTCACACGACGACTCGATACGCGCCTGTTACCCCGCTCGAGGTCAAAA    308
FG-030              ATCATTCGAATCGCCCTCACACGACGACTCGATACGCGCCTGTTACCCCGCTCGAGGTCAAAA    308
FG-039              ATCATTCGAATCGCCCTCACACGACGACTCGATACGCGCCTGTTACCCCGCTCGAGGTCAAAA    314
FG-043              ATCATTCGAATCGCCCTCACACGACGACTCGATACGCGCCTGTTACCCCGCTCGAGGTCAAAA    307
FG-044              ATCATTCGAATCGCCCTCACACGACGACTCGATACGCGCCTGTTACCCCGCTCGAGGTCAAAA    307
FG-057              ATCATTCGAATCGCCCTCACACGACGACTCGATACGCGCCTGTTACCCCGCTCGAGGTCAAAA    311
FG-078              ATCATTCGAATCGCCCTCACACGACGACTCGATACGCGCCTGTTACCCCGCTCGAGGTCAAAA    309
FG-080              ATCATTCGAATCGCCCTCACACGACGACTCGATACGCGCCTGTTACCCCGCTCGAGGTCAAAA    308
FG-082              ATCATTCGAATCGCCCTCACACGACGACTCGATACGCGCCTGTTACCCCGCTCGAGGTCAAAA    313
FG-086              ATCATTCGAATCGCCCTCACACGACGACTCGATACGCGCCTGTTACCCCGCTCGAGGTCAAAA    309
FG-087              ATCATTCGAATCGCCCTCACACGACGACTCGATACGCGCCTGTTACCCCGCTCGAGGTCAAAA    307
FG-092              ATCATTCGAATCGCCCTCACACGACGACTCGATACGCGCCTGTTACCCCGCTCGAGGTCAAAA    310
FG-094              ATCATTCGAATCGCCCTCACACGACGACTCGATACGCGCCTGTTACCCCGCTCGAGGTCAAAA    309
FG-096              ATCATTCGAATCGCCCTCACACGACGACTCGATACGCGCCTGTTACCCCGCTCGAGGTCAAAA    313
FG-098              ATCATTCGAATCGCCCTCACACGACGACTCGATACGCGCCTGTTACCCCGCTCGAGGTCAAAA    308
FG-100              ATCATTCGAATCGCCCTCACACGACGACTCGATACGCGCCTGTTACCCCGCTCGAGGTCAAAA    309
FG-102              ATCATTCGAATCGCCCTCACACGACGACTCGATACGCGCCTGTTACCCCGCTCGAGGTCAAAA    310
FG-103              ATCATTCGAATCGCCCTCACACGACGACTCGATACGCGCCTGTTACCCCGCTCGAGGTCAAAA    310
FG-104              ATCATTCGAATCGCCCTCACACGACGACTCGATACGCGCCTGTTACCCCGCTCGAGGTCAAAA    287
FG-106              ATCATTCGAATCGCCCTCACACGACGACTCGATACGCGCCTGTTACCCCGCTCGAGGTCAAAA    314
FG-107              ATCATTCGAATCGCCCTCACACGACGACTCGATACGCGCCTGTTACCCCGCTCGAGGTCAAAA    313
FG-128              ATCATTCGAATCGCCCTCACACGACGACTCGATACGCGCCTGTTACCCCGCTCGAGGTCAAAA    263
FG-129              ATCATTCGAATCGCCCTCACACGACGACTCGATACGCGCCTGTTACCCCGCTCGAGGTCAAAA    313
FG-130              ATCATTCGAATCGCCCTCACACGACGACTCGATACGCGCCTGTTACCCCGCTCGAGGTCAAAA    309
FG-135              ATCATTCGAATCGCCCTCACACGACGACTCGATACGCGCCTGTTACCCCGCTCGAGGTCAAAA    310
```

```
AF212436.1_Fusar    ATTTTGCGGCTTTGTCGTAAATTTTTTTCTGGTGGGGCTCATACCCCGCCACTCGAGCGACAG    356
AF212444.1_Fusar    ATTTTGCGGCTTTGTCGTAA.TTTTTTTCTGGTGGGGCTCATACCCCGCCACTCGAGCGACAG    356
AJ543588.1_Fusar    ATTTTGCGGCTTTGTCGTAATTTTTTTCCCGGTGGGGCTCATACCCCGCCACTCGAGCGACAG    356
FG-010              ATTTTGCGGCTTTGTCGTAA.TTTTTTTCTGGTGGGGCTCATACCCCGCCACTCGAGCGACAG    376
FG-015              ATTTTGCGGCTTTGTCGTAAATTTTTTTCTGGTGGGGCTCATACCCCGCCACTCGAGCGACAG    375
FG-017              ATTTTGCGGCTTTGTCGTAAATTTTTTTCTGGTGGGGCTCATACCCCGCCACTCGAGCGACAG    354
FG-019              ATTTTGCGGCTTTGTCGTAAATTTTTTTCTGGTGGGGCTCATACCCCGCCACTCGAGCGACAG    373
FG-020              ATTTTGCGGCTTTGTCGTAAATTTTTTTCTGGTGGGGCTCATACCCCGCCACTCGAGCGACAG    372
FG-029              ATTTTGCGGCTTTGTCGTAATTTTTTTCCCGGTGGGGCTCATACCCCGCCACTCGAGCGACAG    371
FG-030              ATTTTGCGGCTTTGTCGTAATTTTTTTCCCGGTGGGGCTCATACCCCGCCACTCGAGCGACAG    371
FG-039              ATTTTGCGGCTTTGTCGTAATTTTTTTCCCGGTGGGGCTCATACCCCGCCACTCGAGCGACAG    377
FG-043              ATTTTGCGGCTTTGTCGTAATTTTTTTCCCGGTGGGGCTCATACCCCGCCACTCGAGCGACAG    370
FG-044              ATTTTGCGGCTTTGTCGTAATTTTTTTCCCGGTGGGGCTCATACCCCGCCACTCGAGCGACAG    370
FG-057              ATTTTGCGGCTTTGTCGTAA.TTTTTTTCTGGTGGGGCTCATACCCCGCCACTCGAGCGACAG    373
FG-078              ATTTTGCGGCTTTGTCGTAATTTTTTTCCCGGTGGGGCTCATACCCCGCCACTCGAGCGACAG    372
FG-080              ATTTTGCGGCTTTGTCGTAATTTTTTTCCCGGTGGGGCTCATACCCCGCCACTCGAGCGACAG    371
FG-082              ATTTTGCGGCTTTGTCGTAATTTTTTTCCCGGTGGGGCTCATACCCCGCCACTCGAGCGACAG    376
FG-086              ATTTTGCGGCTTTGTCGTAATTTTTTTCCCGGTGGGGCTCATACCCCGCCACTCGAGCGACAG    372
FG-087              ATTTTGCGGCTTTGTCGTAATTTTTTTCCCGGTGGGGCTCATACCCCGCCACTCGAGCGACAG    370
FG-092              TTTTTGCGGCTTTGTCGTAA.TTTTTTTCTGGTGGGGCTCATACCCCGCCACTCGAGCGACAG    372
FG-094              ATTTTGCGGCTTTGTCGTAA.TTTTTTTCTGGTGGGGCTCATACCCCGCCACTCGAGCGACAG    371
FG-096              ATTTTGCGGCTTTGTCGTAATTTTTTTCCCGGTGGGGCTCATACCCCGCCACTCGAGCGACAG    376
FG-098              ATTTTGCGGCTTTGTCGTAATTTTTTTCCCGGTGGGGCTCATACCCCGCCACTCGAGCGACAG    371
FG-100              ATTTTGCGGCTTTGTCGTAAATTTTTTTCTGGTGGGGCTCATACCCCGCCACTCGAGCGACAG    372
FG-102              ATTTTGCGGCTTTGTCGTAAATTTTTTTCTGGTGGGGCTCATACCCCGCCACTCGAGCGACAG    373
FG-103              ATTTTGCGGCTTTGTCGTAATTTTTTTCCCGGTGGGGCTCATACCCCGCCACTCGAGCGACAG    373
FG-104              ATTTTGCGGCTTTGTCGTAAATTTTTTTCTGGTGGGGCTCATACCCCGCCACTCGAGCGACAG    350
FG-106              ATTTTGCGGCTTTGTCGTAAATTTTTTTCTGGTGGGGCTCATACCCCGCCACTCGAGCGACAG    377
FG-107              ATTTTGCGGCTTTGTCGTAAATTTTTTTCTGGTGGGGCTCATACCCCGCCACTCGAGCGACAG    376
FG-128              ATTTTGCGGCTTTGTCGTAAATTTTTTTCTGGTGGGGCTCATACCCCGCCACTCGAGCGACAG    326
FG-129              ATTTTGCGGCTTTGTCGTAAATTTTTTTCTGGTGGGGCTCATACCCCGCCACTCGAGCGACAG    376
FG-130              ATTTTGCGGCTTTGTCGTAAATTTTTTTCTGGTGGGGCTCATACCCCGCCACTCGAGCGACAG    372
FG-135              ATTTTGCGGCTTTGTCGTAATTTTTTTCCCGGTGGGGCTCATACCCCGCCACTCGAGCGACAG    373
```

```
AF212436.1_Fusar   GCGTTTGCCCTCTTCCCACAAACCATTCCCTGGGCGCTCATCATCACGTGTCAACCAGTCACT   419
AF212444.1_Fusar   GCGTTTGCCCTCTTCCCACAAACCATTCCCTGGGCGCTCATCATCACGTGTCAACCAGTCACT   419
AJ543588.1_Fusar   GCGTCTGCCCTCTTCCCACAAACCATTCCCTGGGCGCTCATCATCACGTGTCAACCAGTCACT   419
FG-010             GCGTTTGCCCTCTTCCCACAAACCATTCCCTGGGCGCTCATCATCACGTGTCAACCAGTCACT   439
FG-015             GCGTTTGCCCTCTTCCCACAAACCATTCCCTGGGCGCTCATCATCACGTGTCAACCAGTCACT   438
FG-017             GCGTTTGCCCTCTTCCCACAAACCATTCCCTGGGCGCTCATCATCACGTGTCAACCAGTCACT   417
FG-019             GCGTTTGCCCTCTTCCCACAAACCATTCCCTGGGCGCTCATCATCACGTGTCAACCAGTCACT   436
FG-020             GCGTTTGCCCTCTTCCCACAAACCATTCCCTGGGCGCTCATCATCACGTGTCAACCAGTCACT   435
FG-029             GCGTCTGCCCTCTTCCCACAAACCATTCCCTGGGCGCTCATCATCACGTGTCAACCAGTCACT   434
FG-030             GCGTCTGCCCTCTTCCCACAAACCATTCCCTGGGCGCTCATCATCACGTGTCAACCAGTCACT   434
FG-039             GCGTCTGCCCTCTTCCCACAAACCATTCCCTGGGCGCTCATCATCACGTGTCAACCAGTCACT   440
FG-043             GCGTCTGCCCTCTTCCCACAAACCATTCCCTGGGCGCTCATCATCACGTGTCAACCAGTCACT   433
FG-044             GCGTCTGCCCTCTTCCCACAAACCATTCCCTGGGCGCTCATCATCACGTGTCAACCAGTCACT   433
FG-057             GCGTTTGCCCTCTTCCCACAAACCATTCCCTGGGCGCTCATCATCACGTGTCAACCAGTCACT   436
FG-078             GCGTCTGCCCTCTTCCCACAAACCATTCCCTGGGCGCTCATCATCACGTGTCAACCAGTCACT   435
FG-080             GCGTCTGCCCTCTTCCCACAAACCATTCCCTGGGCGCTCATCATCACGTGTCAACCAGTCACT   434
FG-082             GCGTCTGCCCTCTTCCCACAAACCATTCCCTGGGCGCTCATCATCACGTGTCAACCAGTCACT   439
FG-086             GCGTCTGCCCTCTTCCCACAAACCATTCCCTGGGCGCTCATCATCACGTGTCAACCAGTCACT   435
FG-087             GCGTCTGCCCTCTTCCCACAAACCATTCCCTGGGCGCTCATCATCACGTGTCAACCAGTCACT   433
FG-092             GCGTTTGCCCTCTTCCCACAAACCATTCCCTGGGCGCTCATCATCACGTGTCAACCAGTCACT   435
FG-094             GCGTTTGCCCTCTTCCCACAAACCATTCCCTGGGCGCTCATCATCACGTGTCAACCAGTCACT   434
FG-096             GCGTCTGCCCTCTTCCCACAAACCATTCCCTGGGCGCTCATCATCACGTGTCAACCAGTCACT   439
FG-098             GCGTCTGCCCTCTTCCCACAAACCATTCCCTGGGCGCTCATCATCACGTGTCAACCAGTCACT   434
FG-100             GCGTTTGCCCTCTTCCCACAAACCATTCCCTGGGCGCTCATCATCACGTGTCAACCAGTCACT   435
FG-102             GCGTTTGCCCTCTTCCCACAAACCATTCCCTGGGCGCTCATCATCACGTGTCAACCAGTCACT   436
FG-103             GCGTCTGCCCTCTTCCCACAAACCATTCCCTGGGCGCTCATCATCACGTGTCAACCAGTCACT   436
FG-104             GCGTTTGCCCTCTTCCCACAAACCATTCCCTGGGCGCTCATCATCACGTGTCAACCAGTCACT   413
FG-106             GCGTTTGCCCTCTTCCCACAAACCATTCCCTGGGCGCTCATCATCACGTGTCAACCAGTCACT   440
FG-107             GCGTTTGCCCTCTTCCCACAAACCATTCCCTGGGCGCTCATCATCACGTGTCAACCAGTCACT   439
FG-128             GCGTTTGCCCTCTTCCCACAAACCATTCCCTGGGCGCTCATCATCACGTGTCAACCAGTCACT   389
FG-129             GCGTTTGCCCTCTTCCCACAAACCATTCCCTGGGCGCTCATCATCACGTGTCAACCAGTCACT   439
FG-130             GCGTTTGCCCTCTTCCCACAAACCATTCCCTGGGCGCTCATCATCACGTGTCAACCAGTCACT   435
FG-135             GCGTCTGCCCTCTTCCCACAAACCATTCCCTGGGCGCTCATCATCACGTGTCAACCAGTCACT   436
```

```
AF212436.1_Fusar   AACCACCTGTCAATAGGAAGCCGCCGAGCTCGGTAAGGGTTCCTTCAAGTACGCCTGGGTTCT   482
AF212444.1_Fusar   AACCACCTGTCAATAGGAAGCCGCCGAGCTCGGTAAGGGTTCCTTCAAGTACGCCTGGGTTCT   482
AJ543588.1_Fusar   AACCACCTGTCAATAGGAAGCCGCCGAGCTCGGTAAGGGTTCCTTCAAGTACGCCTGGGTTCT   482
FG-010             AACCACCTGTCAATAGGAAGCCGCCGAGCTCGGTAAGGGTTCCTTCAAGTACGCCTGGGTTCT   502
FG-015             AACCACCTGTCAATAGGAAGCCGCCGAGCTCGGTAAGGGTTCCTTCAAGTACGCCTGGGTTCT   501
FG-017             AACCACCTGTCAATAGGAAGCCGCCGAGCTCGGTAAGGGTTCCTTCAAGTACGCCTGGGTTCT   480
FG-019             AACCACCTGTCAATAGGAAGCCGCCGAGCTCGGTAAGGGTTCCTTCAAGTACGCCTGGGTTCT   499
FG-020             AACCACCTGTCAATAGGAAGCCGCCGAGCTCGGTAAGGGTTCCTTCAAGTACGCCTGGGTTCT   498
FG-029             AACCACCTGTCAATAGGAAGCCGCCGAGCTCGGTAAGGGTTCCTTCAAGTACGCCTGGGTTCT   497
FG-030             AACCACCTGTCAATAGGAAGCCGCCGAGCTCGGTAAGGGTTCCTTCAAGTACGCCTGGGTTCT   497
FG-039             AACCACCTGTCAATAGGAAGCCGCCGAGCTCGGTAAGGGTTCCTTCAAGTACGCCTGGGTTCT   503
FG-043             AACCACCTGTCAATAGGAAGCCGCCGAGCTCGGTAAGGGTTCCTTCAAGTACGCCTGGGTTCT   496
FG-044             AACCACCTGTCAATAGGAAGCCGCCGAGCTCGGTAAGGGTTCCTTCAAGTACGCCTGGGTTCT   496
FG-057             AACCACCTGTCAATAGGAAGCCGCCGAGCTCGGTAAGGGTTCCTTCAAGTACGCCTGGGTTCT   499
FG-078             AACCACCTGTCAATAGGAAGCCGCCGAGCTCGGTAAGGGTTCCTTCAAGTACGCCTGGGTTCT   498
FG-080             AACCACCTGTCAATAGGAAGCCGCCGAGCTCGGTAAGGGTTCCTTCAAGTACGCCTGGGTTCT   497
FG-082             AACCACCTGTCAATAGGAAGCCGCCGAGCTCGGTAAGGGTTCCTTCAAGTACGCCTGGGTTCT   502
FG-086             AACCACCTGTCAATAGGAAGCCGCCGAGCTCGGTAAGGGTTCCTTCAAGTACGCCTGGGTTCT   498
FG-087             AACCACCTGTCAATAGGAAGCCGCCGAGCTCGGTAAGGGTTCCTTCAAGTACGCCTGGGTTCT   496
FG-092             AACCACCTGTCAATAGGAAGCCGCCGAGCTCGGTAAGGGTTCCTTCAAGTACGCCTGGGTTCT   498
FG-094             AACCACCTGTCAATAGGAAGCCGCCGAGCTCGGTAAGGGTTCCTTCAAGTACGCCTGGGTTCT   497
FG-096             AACCACCTGTCAATAGGAAGCCGCCGAGCTCGGTAAGGGTTCCTTCAAGTACGCCTGGGTTCT   502
FG-098             AACCACCTGTCAATAGGAAGCCGCCGAGCTCGGTAAGGGTTCCTTCAAGTACGCCTGGGTTCT   497
FG-100             AACCACCTGTCAATAGGAAGCCGCCGAGCTCGGTAAGGGTTCCTTCAAGTACGCCTGGGTTCT   498
FG-102             AACCACCTGTCAATAGGAAGCCGCCGAGCTCGGTAAGGGTTCCTTCAAGTACGCCTGGGTTCT   499
FG-103             AACCACCTGTCAATAGGAAGCCGCCGAGCTCGGTAAGGGTTCCTTCAAGTACGCCTGGGTTCT   499
FG-104             AACCACCTGTCAATAGGAAGCCGCCGAGCTCGGTAAGGGTTCCTTCAAGTACGCCTGGGTTCT   476
FG-106             AACCACCTGTCAATAGGAAGCCGCCGAGCTCGGTAAGGGTTCCTTCAAGTACGCCTGGGTTCT   503
FG-107             AACCACCTGTCAATAGGAAGCCGCCGAGCTCGGTAAGGGTTCCTTCAAGTACGCCTGGGTTCT   502
FG-128             AACCACCTGTCAATAGGAAGCCGCCGAGCTCGGTAAGGGTTCCTTCAAGTACGCCTGGGTTCT   452
FG-129             AACCACCTGTCAATAGGAAGCCGCCGAGCTCGGTAAGGGTTCCTTCAAGTACGCCTGGGTTCT   502
FG-130             AACCACCTGTCAATAGGAAGCCGCCGAGCTCGGTAAGGGTTCCTTCAAGTACGCCTGGGTTCT   498
FG-135             AACCACCTGTCAATAGGAAGCCGCCGAGCTCGGTAAGGGTTCCTTCAAGTACGCCTGGGTTCT   499
```

```
AF212436.1_Fusar    TACTAACATGGCTATCAGACGCTCCCGGTCACCGTG                             644
AF212444.1_Fusar    TACTAACATGGCTCTCAGACGCTCCCGGTCACCGTG                             644
AJ543588.1_Fusar    TACTAACATGGCTATCAGACGCTCCCGGTCACCGTG                             644
FG-010              TACTAACATGGCTCTCAGACGCTCCCGGTCACCGTGATTTCATCAAGAACATGA           682
FG-015              TACTAACATGGCTATCAGACGCTCCCGGTCACCGTGATTTCATCAAGAACATG            680
FG-017              TACTAAC                                                          613
FG-019              TACTAACATGGCTATCAGACGCTCCCGGTCACCGTGATTTCATCAAGAACATGAT          680
FG-020              TACTAACATGGCTATCAGACGCTCCCGGTCACCGTGATTTCATCAAGAACATGA           678
FG-029              TACTAACATGGCTATCAGACGCTCCCGGTCACCGTGATTTCATCAAGAACATGAT          678
FG-030              TACTAACATGGCTATCAGACGCTCCCGGTCACCGTGATTTCATCAAGAACATGAT          678
FG-039              TACTAACATGGCTATCAGACGCTCCCGGTCACCGTGATTTCATCAAGAACATGAT          684
FG-043              TACTAACATGGCTATCAGACGCTCCCGGTCACCGTGATTTCATCAAGAACATG            675
FG-044              TACTAACATGGCTATCAGACGCTCCCGGTCACCGTGATTTCATCAAGAACATGAT          677
FG-057              TACTAACATGGCTCTCAGACGCTCCCGGTCACCGTGATTTCATCAAGAACATG            678
FG-078              TACTAACATGGCTATCAGACGCTCCCGGTCACCGTGATTTCATCAAGAACATGA           678
FG-080              TACTAACATGGCTATCAGACGCTCCCGGTCACCGTGATTTCATCAAGAACATGAT          678
FG-082              TACTAACATGGCTATCAGACGCTCCCGGTCACCGTGATTTCATCAAGAACATGAT          683
FG-086              TACTAACATGGCTATCAGACGCTCCCGGTCACCGTGATTTCATCAAGAACATGA           678
FG-087              TACTAACATGGCTATCAGACGCTCCCGGTCACCGTGATTTCATCAAGAACATGAT          677
FG-092              TACTAACATGGCTCTCAGACGCTCCCGGTCACCGTGATTTCATCAAGAACATG            677
FG-094              TACTAACATGGCTCTCAGACGCTCCCGGTCACCGTGATTTCATCAAGAACATGA           677
FG-096              TACTAACATGGCTATCAGACGCTCCCGGTCACCGTGATTTCATCAAGAACATGA           682
FG-098              TACTAACATGGCTATCAGACGCTCCCGGTCACCGTGATTTCATCAAGAACATGA           677
FG-100              TACTAACATGGCTATCAGACGCTCCCGGTCACCGTGATTTCATCAAGAACATGAT          679
FG-102              TACTAACATGGCTATCAGACGCTCCCGGTCACCGTGATTTCATCAAGAACATGA           679
FG-103              TACTAACATGGCTATCAGACGCTCCCGGTCACCGTGATTTCATCAAGAACATGAT          680
FG-104              TACTAACATGGCTATCAGACGCTCCCGGTCACCGTGATTTCATCAAGAACATGA           656
FG-106              TACTAACATGGCTATCAGACGCTCCCGGTCACCGTGATTTCATCAAGAACATGAT          684
FG-107              TACTAACATGGCTATCAGACGCTCCCGGTCACCGTGATTTCATCAAGAACATGATC         684
FG-128              TACTAACATGGCTATCAGACGCTCCCGGTCACCGTGATTTCATCAAGAACATGATCACTGGTA  641
FG-129              TACTAACATGGCTATCAGACGCTCCCGGTCACCGTGATTTCATCAAGAACATGA           682
FG-130              TACTAACATGGCTATCAGACGCTCCCGGTCACCGTGATTTCATCAAGAACA              675
FG-135              TACTAACATGGCTATCAGACGCTCCCGGTCACCGTGATTTCATCAAGAACATGATC         681

AF212436.1_Fusar
AF212444.1_Fusar
AJ543588.1_Fusar
FG-010
FG-015
FG-017
FG-019
FG-020
FG-029
FG-030
FG-039
FG-043
FG-044
FG-057
FG-078
FG-080
FG-082
FG-086
FG-087
FG-092
FG-094
FG-096
FG-098
FG-100
FG-102
FG-103
FG-104
FG-106
FG-107
FG-128              CT                                                               643
FG-129
FG-130
FG-135
```

Figure S1: The alignments of *TEF-1α* gene sequences of FGSC isolates and standard reference strains. Blue color means the consensus of sequences.
